# Selective benzylic C–H monooxygenation mediated by iodine oxides

**DOI:** 10.3762/bjoc.15.55

**Published:** 2019-03-05

**Authors:** Kelsey B LaMartina, Haley K Kuck, Linda S Oglesbee, Asma Al-Odaini, Nicholas C Boaz

**Affiliations:** 1 Department of Chemistry and Physics, North Central College, 30 N. Brainard Street, Naperville, IL 60540 USA; 2 Department of Chemistry, Frick Chemical Laboratory, Princeton University, Washington Road, Princeton, NJ 08544 USA; 3 Permanent address: Department of Chemistry, North Central College, 30 N. Brainard Street, Naperville, IL 60540 USA; phone: +1-630-637-5187.

**Keywords:** acetoxylation, benzylic, iodate, NHPI, oxidation, radical

## Abstract

A method for the selective monooxdiation of secondary benzylic C–H bonds is described using an *N*-oxyl catalyst and a hypervalent iodine species as a terminal oxidant. Combinations of ammonium iodate and catalytic *N*-hydroxyphthalimide (NHPI) were shown to be effective in the selective oxidation of *n*-butylbenzene directly to 1-phenylbutyl acetate in high yield (86%). This method shows moderate substrate tolerance in the oxygenation of substrates containing secondary benzylic C–H bonds, yielding the corresponding benzylic acetates in good to moderate yield. Tertiary benzylic C–H bonds were shown to be unreactive under similar conditions, despite the weaker C–H bond. A preliminary mechanistic analysis suggests that this NHPI-iodate system is functioning by a radical-based mechanism where iodine generated in situ captures formed benzylic radicals. The benzylic iodide intermediate then solvolyzes to yield the product ester.

## Introduction

The ability to install C–O functionality with high selectivity into a C–H bond is a challenge to synthetic organic chemists. Importantly, this transformation allows for the creation of value-added products from hydrocarbon feedstocks. Benzylic positions are a prevalent example of a functionality whose relatively weak C–H bonds (≈80–90 kcal/mol) make selective functionalization possible [1–2]. The installation of oxygen functionality at benzylic C–H bonds allows for the production of benzylic alcohols, aryl ketones, and aryl carboxylic acids, which are useful chemical building blocks. Strategies that prevent over-oxidation at the target site are particularly useful.

Given the value of the oxidized benzylic products, a substantial amount of effort has been expended in developing ways to selectively transform benzylic C–H bonds to C–O functionalities. Traditionally, this has been accomplished via the treatment of a substrate containing benzylic C–H bonds with acidic sodium dichromate or basic potassium permanganate [3–5]. More recent examples have used metal coordination complexes as catalysts for oxidation [6–20]. Additionally, transition metal-based complexes are widely used in industrial processes as promoters of autoxidation in the functionalization of benzylic C–H bonds of aryl alkanes, such as in the synthesis of terephthalic acid from *p*-xylene [21].

Metal-free oxidants have been used in benzylic C–H to C–O functionalization. Specifically, the use of hypervalent iodine oxidants to mediate benzylic C–H oxidation is one area experiencing a surge of interest [22–33]. Nonmetal-based benzylic oxidations have also been mediated by species including, but not limited to, electron deficient quinones, photoexcited organic dyes and transition metal complexes, hypohalous acids, and persulfate anions [12,34–40].

An important class of catalyzed benzylic C–H to C–O transformations are those catalyzed by *N*-oxyl radicals. Specifically, *N*-oxyl radical catalysts based upon the *N*-hydroxyphthalimide (NHPI) scaffold have been intensely studied for their ability to mediate hydrogen atom abstraction using a terminal oxidant of molecular oxygen [40–45]. NHPI has also been used in the effective C–H to C–O functionalization of benzylic positions using oxidants other than molecular oxygen including NO and HNO_3_ [46–48]. In the work that follows, we report the selective monooxygenation of secondary benzylic C–H bonds of simple aryl alkanes using a combination of iodate and catalytic *N*-hydroxyphthalimide (NHPI), which complements the selectivity and capabilities of existing published work.

## Results and Discussion

We have recently reported a system of simple combinations of an iodine (III, V, or VII) oxide with a catalytic amount of chloride for the direct oxygenation of methane to its methyl ester [49–50]. Mechanistic studies of this system have indicated that these chloride-iodate oxidations occur via a radical pathway mediated in part by chlorine radicals [51]. While the chloride-iodate system was effective in the functionalization of light hydrocarbons and certain model compounds, it exhibited poor functional group tolerance. Oxidation of complex hydrocarbons led to a mixture of intractable products, which were in many cases polyfunctionalized. Additionally, substrates containing arene rings were shown to inhibit aliphatic C–H functionalization via electrophilic trapping of reactive halogen species to form aryl chlorides and iodides.

**Figure 1:**
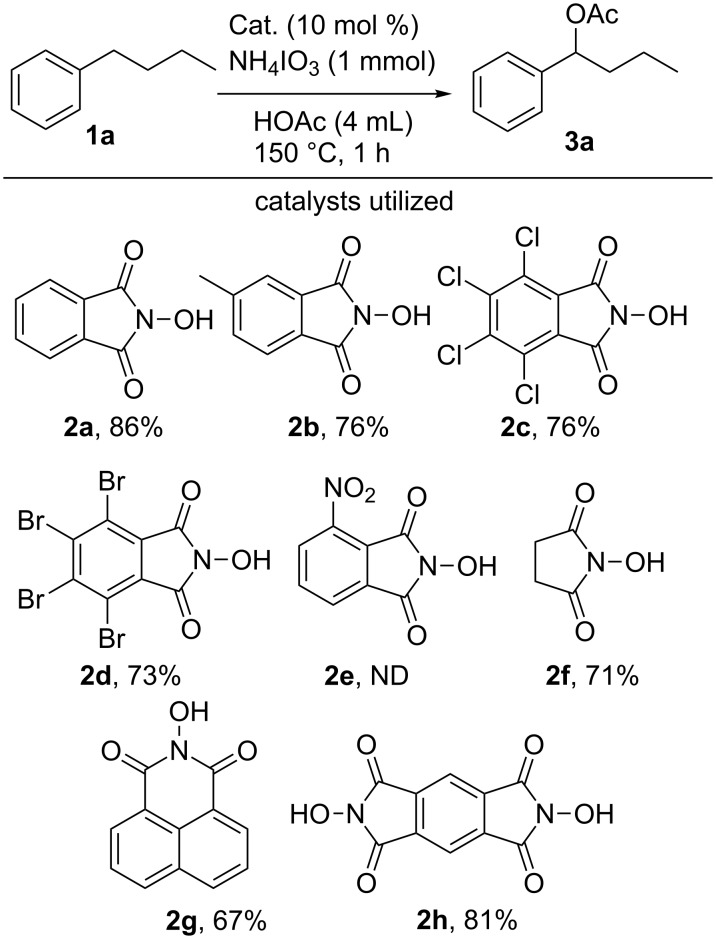
Catalyst optimization for the monooxidation of *n*-butylbenzene mediated by the iodate anion.

Utilizing NHPI as a less reactive hydrogen atom abstraction catalyst than the chlorine radical allowed for the more selective tertiary acetoxylation of adamantane [51]. Nitroxyl radicals formed from species such as NHPI are well studied H-atom abstraction catalysts often used in the functionalization of hydrocarbons [41]. Building upon the ability to selectively acetoxylate the tertiary position of adamantane, we sought to apply this approach to the selective acetoxylation of benzylic C–H bonds. As shown in Figure 1, the use of ammonium iodate in combination with an NHPI-type catalyst yielded efficient benzylic oxidation of *n*-butylbenzene (**1a**) to 1-phenylbutyl acetate (**3a**) with a concomitant reduction of iodate to iodine.

The nature of the H-atom abstraction catalyst was then varied in an effort to increase the yield of compound **3a** upon oxidation of *n*-butylbenzene. The presence of moderate electron withdrawing or donating substituents (chlorine, bromine, or methyl, respectively) slightly lowered the yield when compared to the parent NHPI-type catalyst (catalysts **2b**,**d**). On the other hand, the use of the strongly electron-withdrawing 3-nitro-*N*-hydroxyphthalimide (**2e**) yielded no reaction, returning only starting material. The lack of iodine production upon reaction with **2e** as the catalyst suggests that the strongly electron withdrawing nitro substituent made the catalyst difficult to oxidize under the reaction conditions. While NHPI (**2a**) shows an *E*_1/2_ of 1.065 V the corresponding *E*_1/2_ of 3-nitro-*N*-hydroxyphthalimide (**2e**) is higher at 1.135 V versus Ag/AgNO_3_ [52]. *N*-Hydroxysuccinimide (**2f**) and *N-*hydroxynapthylimide (**2g**) were both able to catalyze the oxidation of *n*-butylbenzene, albeit in slightly lower yield than NHPI. *N,N’*-dihydroxypyromellitimide (catalyst **2h**), which was used as a successful catalyst in the C–H fluorination of benzylic hydrocarbons [53], was also able to catalyze the reaction yielding compound **3a** in 81% yield.

The NHPI-catalyzed oxidation of *n*-butylbenzene (**1a**) was shown to be compatible with a variety of iodine(V) and iodine(VII) oxidants. As shown in Table 1, the use of potassium periodate as the terminal oxidant in the NHPI-catalyzed C–H acetoxylation reaction yielded the ester product **3a** in moderate (42%) yield. This contrasts with orthoperiodic acid (H_5_IO_6_), which yielded only electrophilic arene iodination. The use of alkali metal iodate salts such as lithium, sodium, and potassium iodate as the terminal oxidant yielded **3a** in moderate to good yield (36–84%). Reactions utilizing iodic acid (HIO_3_) and calcium iodate were relatively low-yielding, similar to what was observed in the related chloride-catalyzed methane oxidation system [49]. The oxidant producing the highest yield of **3a**, 86%, was ammonium iodate. Moreover, deviation from the initial 1:1 ratio of oxidant to substrate was shown to significantly reduce the yield of monooxygenated product **3a**. A combination of iodate anion and NHPI has previously been utilized in the oxidation of C–H bonds. Minakata and co-workers recently reported a combination of catalytic NHPI and iodic acid mediated the hydroxylation or amidation of tertiary C–H bonds using either wet nitromethane or dry acetonitrile, respectively [54–55].

**Table 1:** Oxidant optimization for the NHPI-catalyzed monooxidation of *n*-butylbenzene (**1a**).

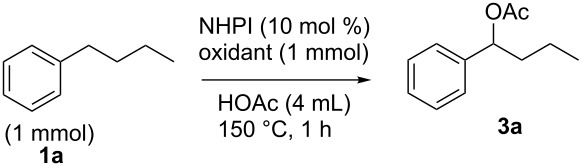		

Oxidant	Iodine oxidation state	Yield^a^

KIO_4_	+7	42%
H_5_IO_6_	+7	Trace
LiIO_3_	+5	36%
NaIO_3_	+5	84%
KIO_3_	+5	83%
Ca(IO_3_)_2_	+5	17%
HIO_3_	+5	43%
NH_4_IO_3_	+5	86%
NH_4_IO_3_ (0.9 mmol)	+5	73%
NH_4_IO_3_ (1.1 mmol)	+5	74%

^a^Yields were determined by gas chromatography relative to an internal standard of dodecane. All reported yields are the average of 3 individual reactions.

**Figure 2:**
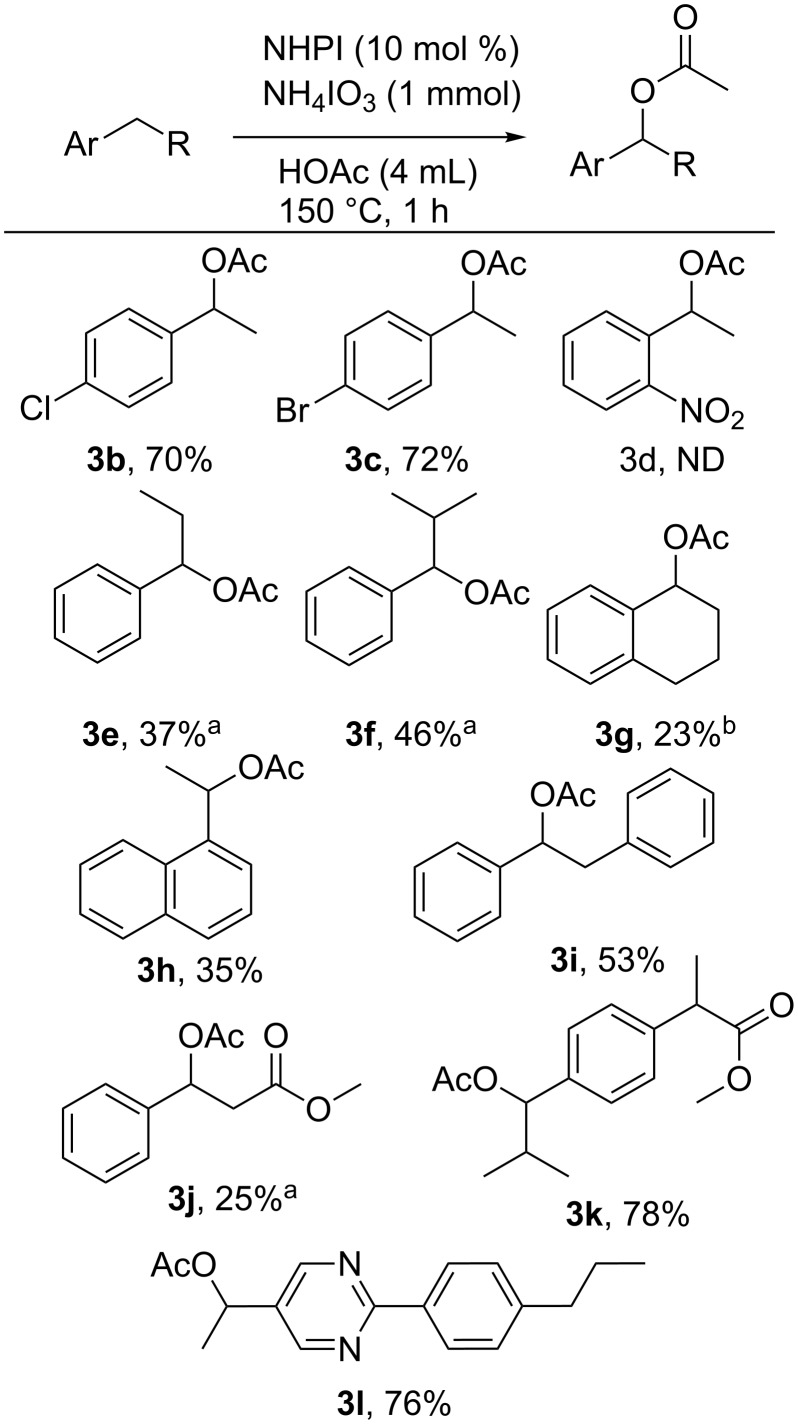
NHPI-catalyzed oxidation of secondary benzylic C–H bonds mediated by iodine(V). ^a^100 °C for 18 h; ^b^60 °C for 18 h.

The substrate scope of the developed benzylic C–H monooxygenation reaction was examined via the oxidation of substrates containing benzylic C–H bonds as well as other functional groups. The optimized conditions developed for the acetoxylation of **1a** were used in the effective oxidation of other substrates containing secondary benzylic C–H bonds. As shown in Figure 2, treatment of *p*-bromo or *p*-chloroethylbenzene with the optimized conditions cleanly yielded monooxidized acetate esters **3b** and **3c** in 70 and 72% yield, respectively. Bibenzyl and 1-ethylnaphthalene were also oxidized in reasonable yield to acetate esters **3i** and **3h**. The oxidation of the biologically active ibuprofen methyl ester yielded the secondary acetate **3k** in 78% yield. Finally, the oxidation of 5-ethyl-2-(4-propylphenyl)pyrimidine to acetate ester **3l** in 76% yield indicates that the developed catalytic system is tolerant of nitrogen-containing heterocycles. This reaction shows remarkable selectivity for the alkyl chain appended to the pyrimidine ring as opposed to the propyl group attached to the benzene ring. We propose that the methylene group adjacent to the pyrimidine ring possesses lower bond strength C–H bonds than those adjacent to the benzene ring. This difference in bond strength is illustrated when comparing the benzylic C–H bonds of toluene to that of 2-methylpyridine (89.7 versus 87.2 kcal/mol, respectively) [56]. Moreover, the electron withdrawing nature of the pyrimidine nitrogen atoms will affect the p*K*_a_ of the adjacent secondary benzylic C–H bonds. The hydrogen atom abstraction from this position would then be influenced by the p*K*_a_ of the C–H bond via a proton coupled electron transfer (PCET) type mechanism.

**Figure 3:**
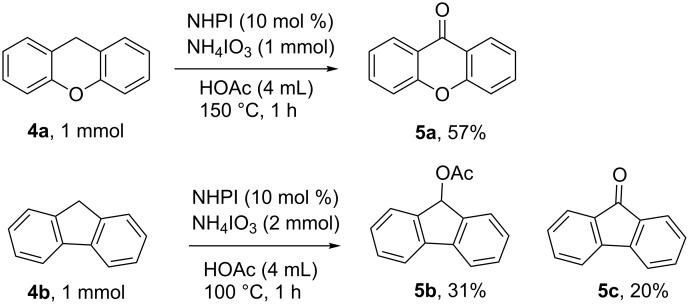
NHPI-catalyzed oxidation of di-benzylic C–H bonds mediated by iodate.

Additionally, as shown in Figure 2, 1,2,3,4-tetrahydronaphthalene was functionalized in poor yield (23%) to its acetate **3g** if exposed to reaction conditions at lower temperatures (60 °C) than were used for other substrates. At 100 or 150 °C, only aromatization was observed, suggesting that monooxygenated products are able to react further. We propose that acetoxylated tetrahydronaphthalene eliminates acetic acid to yield dihydronaphthalene, which can be further oxidized by two electrons to yield the stable naphthalene ring. Minisci and co-workers also reported aromatization upon oxidation of tetrahydronaphthalene using an acetoxylation system similar to that reported in this work [57]. While the methodology described in this work is tolerant of molecular functionality with moderate oxidative stability such as esters and nitrogen-containing heterocycles, it shows incompatibility with functional groups such as alkenes, phenols, phenyl ethers, and alcohols, which are readily oxidized.

The dibenzylic C–H bonds of fluorene and xanthene were also able to be functionalized. Xanthene (**4a**) was over-oxidized to the ketone (xanthone, **5a**) in 57% yield (Figure 3). Similarly, fluorene was oxidized to a mixture of 9-fluorenyl acetate (**5b**, 31%) and fluorenone (**5c**, 20%). The same mixture of acetate and ketone is observed at a higher temperature (150 °C), but the overall yield was lower. It is proposed that the stabilizing presence of two benzene rings on the monooxidized products activates the remaining benzylic C–H bond in a way that encourages overoxidation.

**Figure 4:**
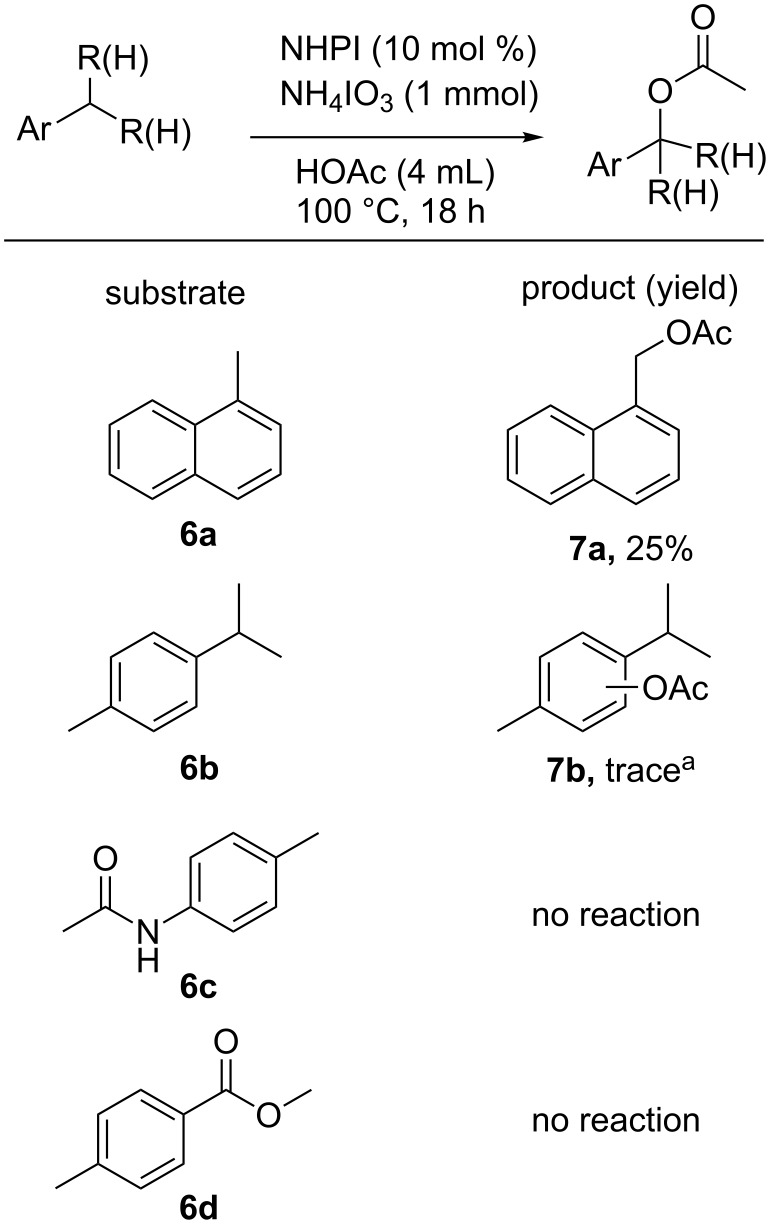
NHPI-catalyzed oxidation of substrates containing primary and tertiary benzylic C–H bonds. ^a^Reaction performed using NaIO_3_ (1 mmol) at 100 °C for 18 h. Product detected using GC–MS.

The oxidation of 1-methylnaphthalene, (**6a**) as shown in Figure 4, indicates that functionalization of substrates containing primary benzylic C–H bonds was also possible using the NHPI-iodate system. Such functionalization, however, appears to be more limited in scope and yield than that of secondary benzylic C–H bonds in that 4’-methylacetanilide (**6c**), and methyl 4-methylbenzoate (**6d**) were unable to be functionalized. It is postulated that the increased bond strength of primary benzylic C–H bonds when compared to secondary benzylic C–H bonds makes them less able to be functionalized in this catalytic system [56]. A similar preference for the oxidation of secondary benzylic positions over primary benzylic positions was recently reported by Noël and co-workers in the aerobic oxidation of C–H bonds catalyzed by decatungstate [58]. Substrates containing tertiary C–H bonds, such as 1-isopropyl-4-methylbenzene (**6b**), were essentially unreactive under the developed conditions, returning starting alkyl arene with only a trace of acetoxylated product **7b** (see Supporting Information File 1, Figure S11) [59]. Previous work on the oxidation of tertiary benzylic C–H bonds indicates that in other catalytic systems, NHPI is able to mediate the abstraction of a hydrogen atom from a tertiary benzylic position. The identity and selectivity of the product produced, however, is strongly influenced by the reaction conditions. Works by Ishii and co-workers on the aerobic oxidation of cumene in acetic acid using catalytic NHPI and cobalt(II), resulted in a mixture of 2-phenyl-2-propanol, acetophenone, and phenol [60–61]. This lack of selectivity in the product was related in part to the propensity of the cumene hydroperoxide intermediate to decompose into phenol and acetone under acidic conditions [62]. Use of a less acidic solvent and lower reaction temperature drastically increased the selectivity for 2-phenyl-2-propanol in the NHPI-catalyzed oxidation of cumene [57]. The reason why tertiary benzylic C–H bonds, which are weaker than secondary and primary benzylic C–H bonds, are unreactive in the system described within this work is not immediately clear, however, increased steric constraints at a tertiary carbon may play a role.

The catalytic system proposed herein is related to work published by Minisci et al. that details the use of a catalytic system using HNO_3_, dioxygen, iodine and NHPI in the acetoxylation of C–H bonds [47]. Given this similarity, we believe that the two systems are operating by similar mechanisms. In the Minisci system, a phthalimide *N*-oxyl (PINO) radical, which is formed by the oxidation of NHPI by HNO_3_ abstracts a C–H bond from a substrate yielding an organic radical. The organic radical is then trapped by iodine to yield the alkyl iodide which is subsequently functionalized by acetic acid to yield the acetate product.

**Figure 5:**
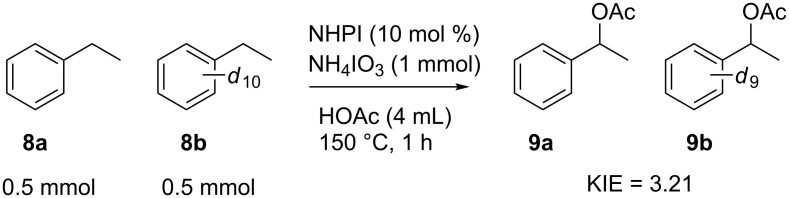
Competitive deuterium KIE for the oxidation of ethyl benzene by the NHPI-iodate system.

To examine if the catalytic system proposed in this work is proceeding via a similar mechanism we examined several aspects of the NHPI-iodate system. A substrate deuterium kinetic isotope effect (KIE) study indicated that the benzylic C–H bond is broken more rapidly than the C–D bond of the deuterated substrate. Catalytic oxidation of a stoichiometric mixture of proteo and perdeutero ethylbenzene (**8a**,**b**) afforded a deuterium KIE of 3.21 ± 0.08 (Figure 5). The magnitude of this value indicates a primary KIE, which is observed with other reported deuterium KIE values of benzylic C–H scission via PINO radicals. Deuterium KIE values for hydrogen atom abstraction by the PINO radical vary based upon conditions such as temperature and solvent [63–65]. Ishii and co-workers have shown that catalytic oxidation of ethylbenzene in acetic acid solvent via PINO radical catalysis yields a competitive deuterium KIE of 3.8, similar to the system described herein [60].

**Figure 6:**
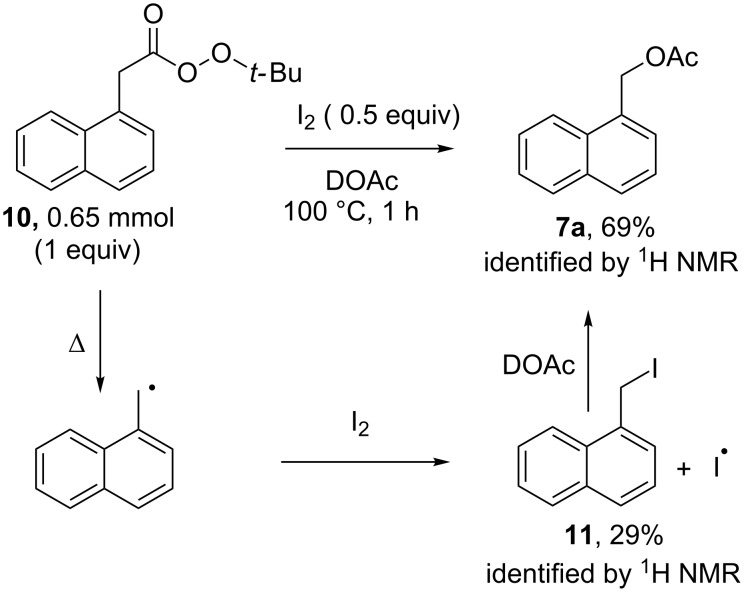
Pyrolysis of an acyl perester in the presence of molecular iodine.

The existence of a primary KIE suggests the cleavage of the benzylic C–H bond and formation of a benzylic radical during or prior to the rate determining step of the reaction. Such a mechanism is in line with other C–H oxidations catalyzed by NHPI [41,53,66–67]. It is proposed that formed aliphatic radicals are trapped by molecular iodine which is produced under catalytic conditions via the reduction of iodate. While iodine is produced under experimental conditions via the reduction of iodate, it is not entirely clear what the origin of all the electrons for this process is given that the benzylic oxidation is only a net 2 electron process. Previous research shows that the rate of radical trapping by molecular iodine nears diffusion control, similar to that of diatomic oxygen [68–70]. This process of radical trapping was probed through the pyrolysis of *tert*-butyl 2-(naphthalen-1-yl)ethaneperoxoate (**10**), a benzylic radical precursor [71], in the presence of molecular iodine. Heating of this acyl perester at 100 °C for 1 hour in the presence of molecular iodine yielded acetate ester **7a** in 69% yield and the benzylic iodide **11** in 29% yield (Supporting Information File 1, Figures S3 and S4). When heated, the perester will decompose to form the relatively stable benzylic radical. Such a radical is then trapped by molecular iodine to form the benzylic iodide, which is subsequently solvolyzed to yield the acetate ester product (Figure 6). This mechanism of radical capture by iodine is similar to what we recently proposed for the chloride-iodate mediated oxidation of methane and in line with similar processes described in the literature, including the catalytic system described by Minisci [47,51,54–55,72–73]. Minakata and co-workers reported that a similar catalytic cycle using iodic acid and NHPI oxidation of formed alkyl iodide intermediate to an iodine(III) species was necessary for the conversion to a substituted product [54–55]. While we cannot rule out that oxidation of formed benzylic iodide intermediates occurs in the production of benzylic acetate the production of ester product without the need for oxidation indicates that oxidation may not be necessary.

**Figure 7:**
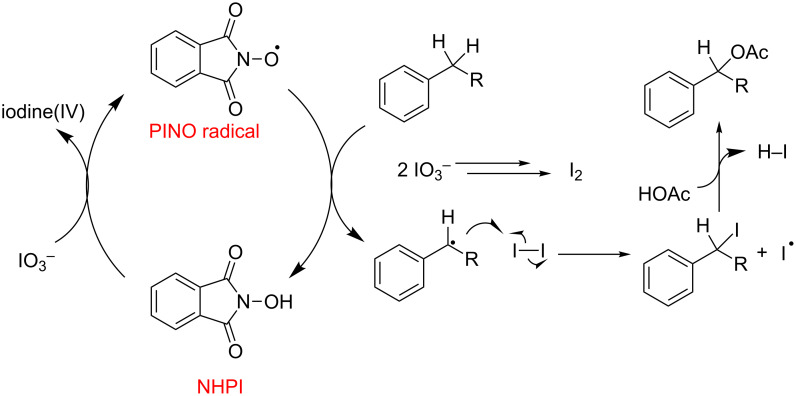
Proposed mechanism for the selective monooxygenation of benzylic C–H bonds.

As shown in Figure 7, a radical-based catalytic cycle is proposed for the NHPI-catalyzed oxidation of benzylic C–H bonds. NHPI is oxidized by one electron by iodate to form the well-characterized PINO radical. The PINO radical, which forms an OH bond of 88 kcal/mol upon hydrogen abstraction (Figure 7) [63], will preferentially attack the weaker C–H bonds at the benzylic carbon, forming a relatively stable benzylic radical, rather than C–H bonds with stronger bond dissociation enthalpy values. The formed benzylic radicals are then captured by I_2_, generated in situ by the reduction of iodate. The benzylic iodide formed upon trapping is expected to be reactive, readily converting to the corresponding ester in carboxylic acid media.

## Conclusion

A metal-free method for the selective monooxygenation of secondary benzylic C–H bonds using a combination of iodate and NHPI was developed. The oxidation of *n*-butylbenzene with stochiometric iodate and catalytic NHPI resulted in the clean conversion of substrate to 1-phenylbutyl acetate (**3a**) in 86% yield. The scope of this reaction appears to be tolerant of various functional groups including halide groups, esters, and nitrogen-containing heterocycles, yielding monoxidized acetate products in moderate to good yields. While the reaction conditions were effective in the oxidation of secondary benzylic C–H bonds, the reaction was less effective in the oxidation of primary benzylic C–H bonds. Moreover, substrates with tertiary benzylic C–H bonds were unreactive despite having a weaker C–H bond strength. Preliminary mechanistic studies indicate that the reaction occurs via H-atom abstraction mediated by the PINO radical followed by trapping with molecular iodine. The formed benzylic iodide is then solvolyzed to yield the final benzylic acetate ester.

## Supporting Information

File 1 All experimental procedures, analytical data, and copies of ^1^H NMR spectra of all studied compounds.
