# Supplementary material for: Selective benzylic C–H monooxygenation mediated by iodine oxides
Source: Beilstein J Org Chem. 2019 Mar 5;15:602–9. doi: 10.3762/bjoc.15.55 (PMC6423598; doi:10.3762/bjoc.15.55)
Supplement: File 1 — All experimental procedures, analytical data, and copies of 1H NMR spectra of all studied compounds. [file Beilstein_J_Org_Chem-15-602-s001.pdf]

BEILSTEIN JOURNAL OF ORGANIC CHEMISTRY

Supporting Information

for

Selective benzylic C–H monooxygenation mediated by iodine oxides

Kelsey B. LaMartina, Haley K. Kuck, Linda S. Oglesbee, Asma Al-Odaini and Nicholas C. Boaz

Beilstein J. Org. Chem. **2019,** *15,* 602–609. doi:10.3762/bjoc.15.55

All experimental procedures, analytical data, and copies of ^{1}H NMR spectra of all studied compounds

License and Terms: This is a supporting information file under the terms of the Creative Commons Attribution License (http://creativecommons.org/licenses/by/4.0). Please note that the reuse, redistribution and reproduction in particular requires that the authors and source are credited. The license is subject to the *Beilstein Journal of Organic Chemistry* terms and conditions: (https://www.beilstein-journals.org/bjoc)

Contents

General experimental details-S1

Oxidation of *n*-butylbenzene using *N*-Oxyl catalysts-S1

Intermolecular kinetic isotope measurements-S1

Synthesis and subsequent pyrolysis of *tert*-butyl-2-(napthalen-1-yl)ethaneperoxoate-S2

General methods for catalytic oxidation of benzylic C-H bonds-S7

Experimental methods of characterization data for benzylic esters produced catalytically-S8

Characterization of a mixture of fluoreneone and fluoren-9-acetate-S13

NMR spectra for esters produced catalytically-S21

References-S38

General experimental details

Caution: Many of the reagents and conditions set forth in this paper are potentially dangerous. Please ensure that proper precautions are taken and literature consulted when working with strong oxidants (high valent iodine oxides) and strong acids.[1]

Unless indicated otherwise, all reactions were carried out under ambient atmosphere. *N*-butylbenzene, 4-chloroethylbenzene, 4-bromoethylbenzene, 2-nitroethylbenzene, bibenzyl, 1-ethylnapthylene, and fluorene were purchased from Sigma-Aldrich and used without further purification. Xanthene was also purchased from Sigma-Aldrich and was re-crystallized from isopropanol prior to use. Ibuprofen (sodium salt) was purchased from Sigma-Aldrich and converted to the methyl ester via treatment with $SOCl_2$ in methanol according to literature methods [2]. Glacial acetic acid, chloroform, hexanes, and ethyl acetate were purchased from Fischer Scientific and used without further purification. 2-(4-ethylphenyl)-5-*n*-propyl)pyrimidine was purchased from Alpha Aeser and used without further purification. All iodate and periodate oxidants were purchased from VWR and used without further purification. Silica gel was purchased from Teledyne-Isco as 12-gram single-use columns or from Biotage as 10-gram single use columns. Deuterated chloroform was purchased from Cambridge Isotopes or Sigma Aldrich and were stored over activated 3-angstrom molecular sieves. Deuterated acetic acid was purchased from Sigma-Aldrich and used without further purification.

The *n*-oxyl catalysts *N*-hydroxyphthalimide (**2a**), and *N*-hydroxysuccinimide (**2f**) were purchased from Sigma-Aldrich and used without further purification. Tetrachloro-*N*-hydroxyphthalimide (**2c**), tetrabromo-*N*-hydroxyphthalimide (**2d**), 3-methyl-*N*-hyroxyphthalimide (**2b**), 3-nitro-*N*-hydroxyphthalimide (**2e**), *N*-hydroxynapthalimide (**2g**), and *N,N*'-dihydroxypyromellitimide (**2h**) were synthesized via condensation with their respective anhydride precursors using established literature procedures [3-6].

GC–MS analysis was performed using an Agilent Technologies 7890A gas chromatograph or a Perkin Elmer Clarius 580 gas chromatograph equipped with a fused silica column and electron impact mass analyzer. High resolution electrospray ionization mass spectral analysis was performed using an Agilent 6220 Accurate-Mass Time-of-Flight LC/MS in positive ion mode. NMR analysis was performed using a Bruker 300 Avance Spectrometer or a Bruker 500 Avance Spectrometer equipped with a cryo-cooled multinuclear probe. Flash chromatography was performed on Teledyne-Isco flash chromatography system or a Biotage flash chromatography system equipped with a UV–vis detector and an automated fraction collector.

Oxidation of *n*-butylbenzene using *N*-oxyl catalysts

In a typical reaction, an 8 mL microwave vial equipped with a stirbar was charged with ammonium iodate (1 mmol), *n*-butylbenzene (1 mmol), and catalyst (0.10 mmol). Glacial acetic acid (4 mL) was then added and the reaction vial sealed with a PTFE lined crimp cap. The vials were then heated in an oil bath at 150 °C for one hour with vigorous stirring (1150 RPM). After one hour, the vial was allowed to cool to room temperature at which point the reaction mixture is added to 5 mL of chloroform containing 1-octyl trifluoroacetate (1 mmol), added as an internal standard. The mixture was then extracted 2× with 10 mL of water and the organic washings dried over $MgSO_4$. The yield of 1-phenylbutyl acetate was determined by GC–MS relative to the added internal standard. Each oxidation was repeated a minimum of 3× with the average of all reactions reported.

Intermolecular KIE Measurements

Intermolecular KIE values were determined by performing the functionalization of a 1:1 stoichiometric ratio of proteo (0.5 mmol) and perdeutero (0.5 mmol) ethylbenzene. Briefly, an 8 mL microwave vial equipped was charged with proteo and perdeutero ethylbenzene 8a-b. NHPI (0.1 mmol), and ammonium iodate (0.2 mmol) were then added to the reaction mixture. Only 20 mol. % of oxidant relative to the total molar amount of substrate was added ensure that conversion was kept below 20%. Finally the reaction vial was charged with acetic acid solvent (4 mL) and the vial sealed with a crimp cap. The reaction was then heated to 150 °C for 1 hour using vigorous stirring (1150 RPM). After one hour, the vial was allowed to cool to room temperature at which point the reaction mixture was added to 5 mL of chloroform containing 1-octyl trifluoroacetate (1 mmol), added as an internal standard. The mixture was then extracted 2X with 10 mL of water and the organic washings dried over $MgSO_4$. The yield of proteo and perdeutero 1-phenethyl acetate 9a-b was then determined via GC-MS relative to the added internal standard. The intermolecular deuterium KIE was then obtained by determining the ratio of proteo product to deutero product. The deuterium KIE ratio reported is the average of 3 individual experiments.

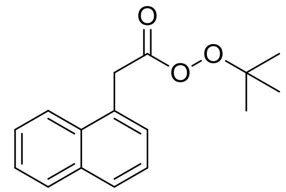

Synthesis of *tert*-butyl 2-(napthalen-1-yl)ethaneperoxoate (10)
The title compound was synthesized using the method of Sammis et al. [7]. Briefly, a 100 mL roundbottom flask, equipped with a stir bar, was charged with 1-napthaleneacetic acid (12.2 mmol) and methylene chloride (12.2 mL). *N,N*-Dimethylaminopyridine (DMAP) (1.22 mmol) was then added and the solution was cooled to 0 °C. Tert-butyl hydroperoxide (12.81 mmol, 5.5 M in decane) was then added dropwise to the stirring solution and allowed to stir for five minutes. *N*,*N'*-dicyclohexylcarbodiimide (DCC) (13.42 mmol, 0.2 M in methylene chloride) was then added over 30 minutes. The mixture was then allowed to warm to room temperature and stirred for 18 h. Solids were removed from the mixture via filtration and the solution concentrated in vacuo. The mixture was purified via flash chromatography (silica gel eluting with hexanes/ethyl acetate) to yield the title product as a clear oil (1.96 g, 62% yield) which was stored at 0 °C. The product was characterized via ^{1}H NMR, ^{13}C NMR, and IR spectroscopy.

CAUTION: starting materials and products containing a peroxide moiety can be unstable. Synthetic operations such as removal of solvent under reduced pressure should be performed behind a blast shield.

^{1}H NMR (d_4 Acetic Acid, 300 MHz, δ) 8.05 (d, J = 8.5 hz, 1H), 7.89 (d, J = 8.0 hz, 1H), 7.84 (d, J = 7.46 hz, 1H), 7.62-7.40 (m, 4H), 4.17 (s, 2H), 1.13 (s, 9H) ^{13}C NMR (75 MHz, $CDCl_3$)168.71, 133.82, 131.85, 129.20, 128.80, 128.43, 128.05, 126.53, 125.94, 125.48, 123.61, 83.76, 36.50, 25.99. IR (ATIR) v 3047.65, 2978.58, 2932.14, 1769.92, 1188.99, 1164.60, 1093.87 cm^{-1}. The instability of the internal acyl peroxide precluded the ability to characterize by MS as only decomposed product was observed.

Pyrolysis of *tert*-butyl 2-(napthalen-1-yl)ethaneperoxoate in the presence of iodine

An NMR tube was charged with *tert*-butyl 2-(napthalen-1-yl)ethaneperoxoate (0.066 mmol, 17 mg), iodine (0.033 mmol, 4 mg), cyclopentane (2 μL, internal standard), and acetic acid-d_4 (1 mL). The mixture was analyzed via ^{1}H NMR and then heated at 100 °C for 1 h. After allowing the mixture to cool to room temperature the solution was again analyzed by ^{1}H NMR. Relative to the internal standard of cyclopentane all of the starting material had been consumed and naphthalene-1-ylmethyl acetate 7a had been formed in 68% yield and 1-iodomethylnaphthalene **11** was formed in 30% yield (See Figures S3 and S4).

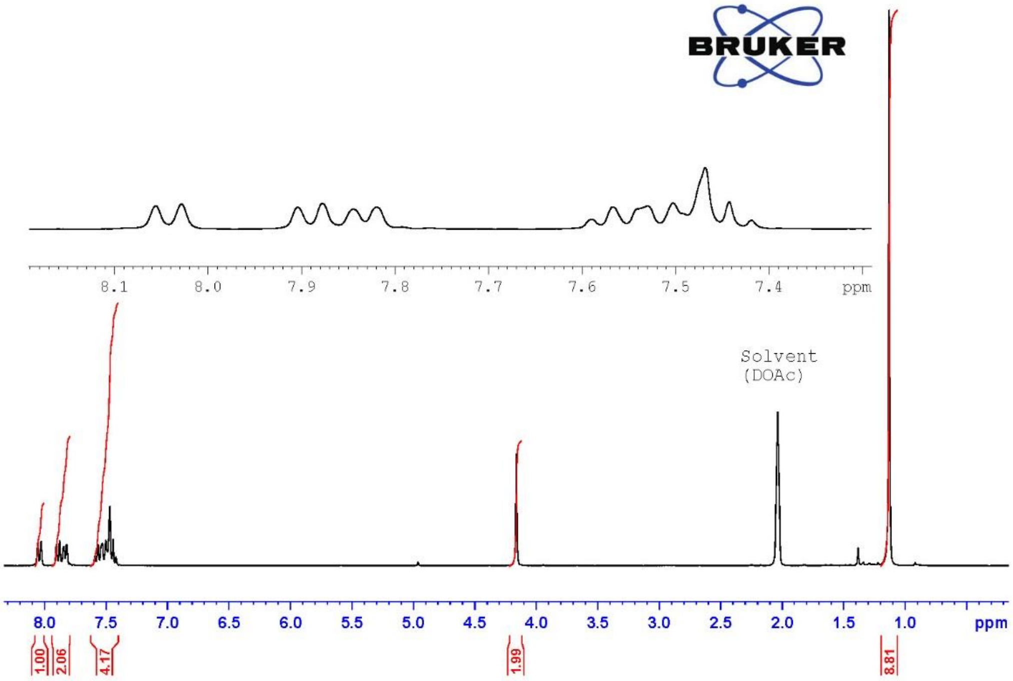

Figure S1-^{1}H NMR of *tert*-butyl 2-(napthalen-1-yl)ethaneperoxoate.

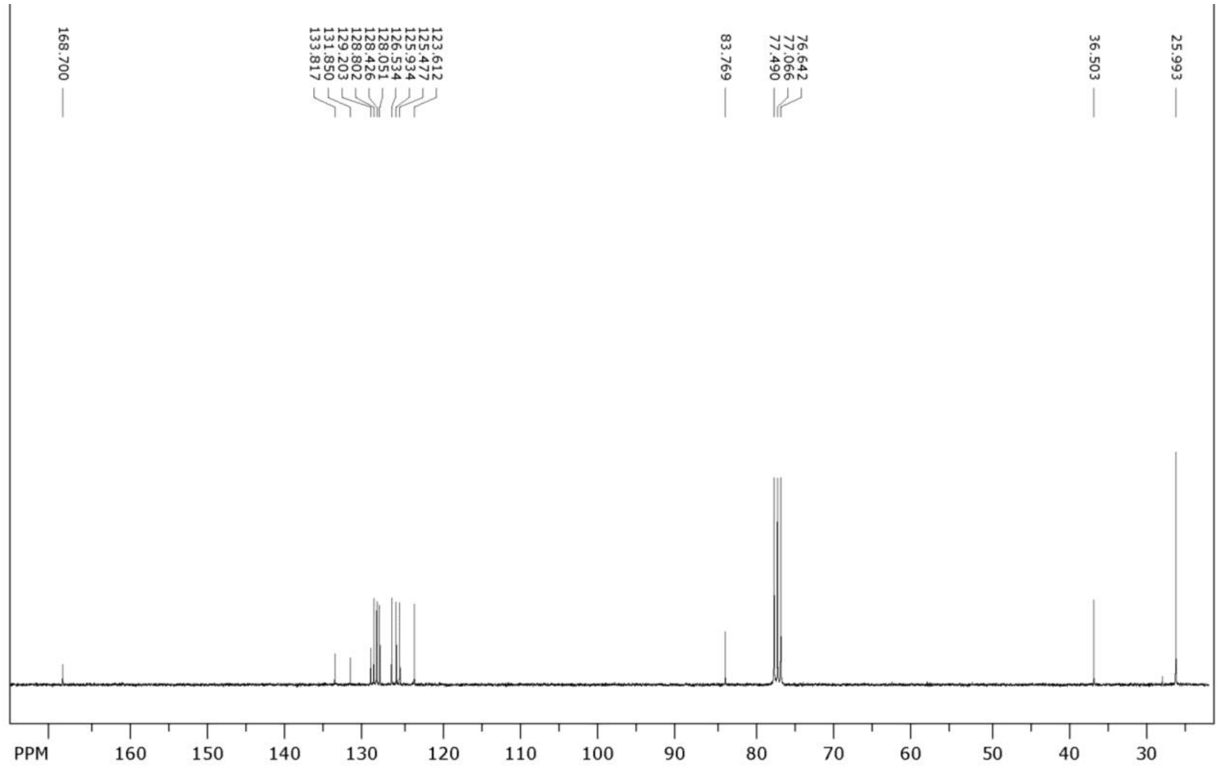

Figure S2- ^{13}C NMR of *tert*-butyl 2-(napthalen-1-yl)ethaneperoxoate.

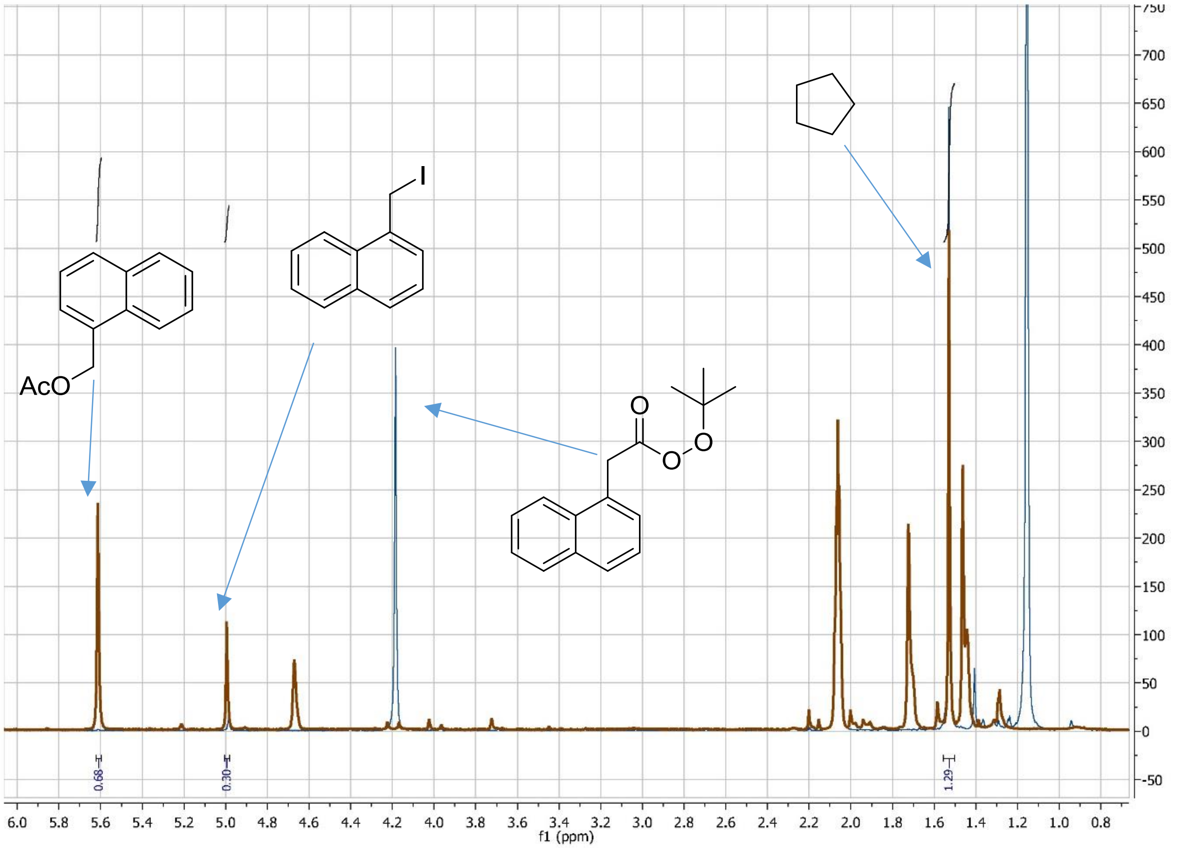

Figure S3. ^{1}H NMR overlay of perester mixture prior to heating (green) and after heating (brown). The perester CH_2 group is set to an integration of 1.00 relative to an internal standard of cyclopentane having an integration of 1.29.

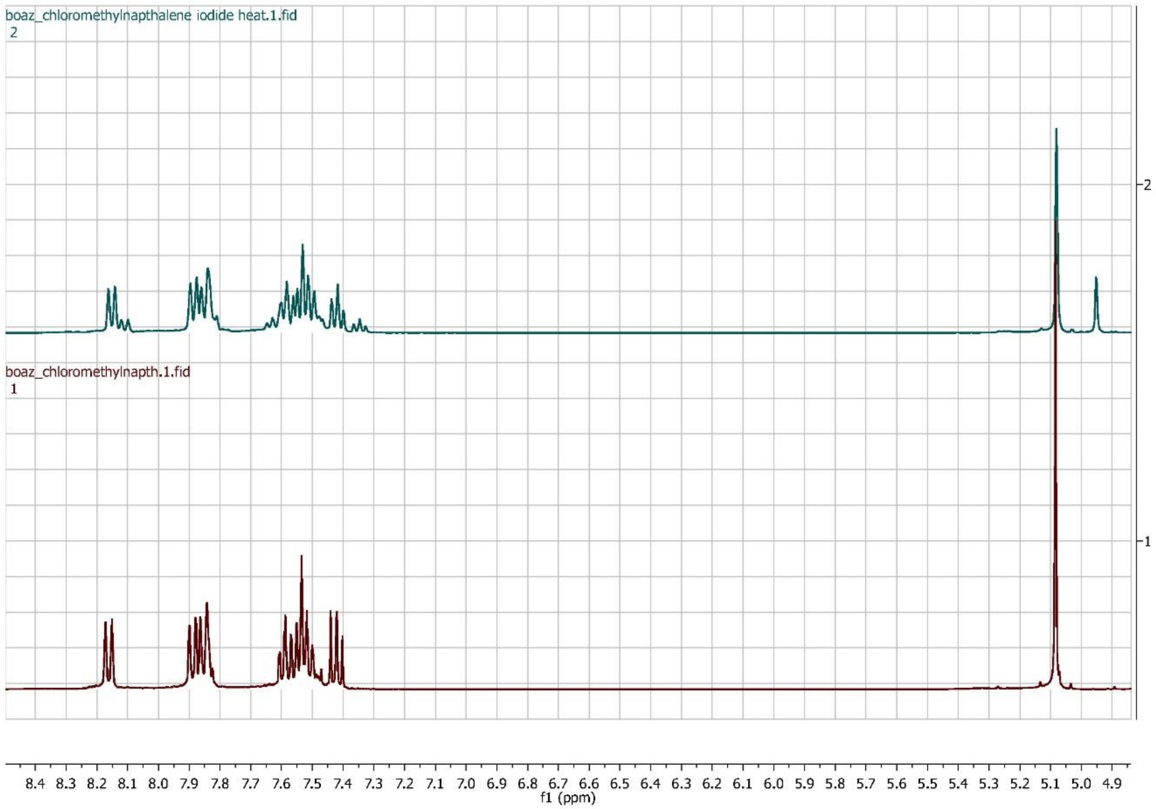

Figure S4. In situ generation of 1-iodomethylnaphthalene **11** as monitored via ^{1}H NMR. (Red) 1-chloromethylnaphthalene in deutero acetic acid. (Green) Addition of sodium iodide and heat (30 °C, 1 h) generate 1-iodomethylnaphthalene in situ.

Catalytic oxidation of benzylic C–H bonds

General procedure A: acetoxylation of substrates with benzylic C–H bonds

An 8 mL microwave vial equipped with a stirbar was charged with ammonium iodate (1 mmol), substrate (1 mmol), and *N*-hydroxyphthalimide (0.10 mmol). Glacial acetic acid (4 mL) was added as solvent and the reaction sealed with a crimp cap. The mixture was stirred at 150 °C for one hour during which time the reaction developed a deep purple color. The reaction was then allowed to cool and then added to 10 mL of $CHCl_3$. The mixture was extracted with 10 mL of saturated sodium metabisulfate, 10 mL of saturated sodium bicarbonate, and 2× with 10 mL of brine. The organic washings were dried over anhydrous $MgSO_4$ and the solvent removed under reduced pressure The crude residue was then purified via flash chromatography on silica gel eluting with a mixture of hexanes and ethyl acetate.

General procedure B: acetoxylation of substrates with benzylic C–H bonds

A 4 mL conical vial equipped with a stirbar was charged with ammonium iodate (2 mmol), substrate (2 mmol), and *N*-hydroxyphthalimide (0.20 mmol). Glacial acetic acid (4 mL) was added as solvent and the reaction sealed with a screwcap vial

equipped with a PTFE septum. The mixture was stirred at 100 °C for 18 hours during which the reaction developed a deep purple color. The reaction was then allowed to cool and then added to 10 mL of $CHCl_3$. The mixture was extracted with 10 mL of saturated sodium metabisulfate, 10 mL of saturated sodium bicarbonate, and 2× with 10 mL of brine. The organic washings were dried over anhydrous $MgSO_4$ and the solvent removed under reduced pressure The crude residue was then purified via flash chromatography on silica gel eluting with a mixture of hexanes and ethyl acetate.

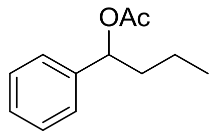

1-Phenylbutyl acetate (3a) was prepared in an 8 mL microwave vial according to general procedure A using *n*-butylbenzene (134 mg, 1 mmol), ammonium iodate (193 mg, 1 mmol), and *N*-hydroxyphthalimide to afford the title complex as a colorless oil (86%, via GC relative to an internal standard of dodecane). The product was characterized via ^{1}H NMR, ^{13}C NMR, IR, and EI-MS and matched spectral data previously reported.[8]

($CDCl_3$, 500 MHz, δ) 7.28 – 7.24 (m, 4H), 7.22 – 7.16 (m, 1H), 5.66 (dd, *J* = 7.8, 6.2 Hz, 1H), 1.98 (s, 3H), 1.82 (dddd, *J* = 13.3, 10.0, 7.7, 5.4 Hz, 1H), 1.66 (ddt, *J* = 13.6, 10.0, 6.0 Hz, 1H), 1.28 (dtdd, *J* = 16.6, 9.0, 4.5, 1.8 Hz, 1H), 1.23 – 1.12 (m, 1H), 0.84 (t, *J* = 7.4 Hz, 3H).^{13}C NMR ($CDCl_3$, 126 MHz, δ) 170.42, 140.87, 128.41, 127.82, 126.53, 75.92, 38.45, 21.31, 18.82, 13.83. IR (ATIR) ν 3035, 2960, 2874, 1732, 1230, 1023, and 698 cm^{-1}. EI-MS $C_{12}H_{16}O_2$ expected 192.12 m/z observed 192.3 m/z.

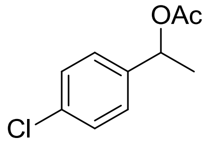

1-(4-Chlorophenyl)ethyl acetate (3b) was prepared in an 8 mL microwave vial according to general procedure A using 4-chloroethylbenzene (141 mg, 1 mmol), ammonium iodate (193 mg, 1 mmol), and *N*-hydroxyphthalimide to afford the title complex as a yellow oil (139 mg, 70%). The product was characterized via ^{1}H NMR, ^{13}C NMR, IR, and EI-MS and the spectra obtained matched previously reported spectral values.[10]

^{1}H NMR ($CDCl_3$, 500 MHz, δ) 7.31-7.25 (m, 4H), 5.81 (q, *J* = 6.6 Hz, 1H), 2.05 (s, 3H), 1.49 (d, *J* = 6.6 Hz, 3H), ^{13}C NMR ($CDCl_3$, 126 MHz, δ) 170.20, 140.20, 133.62, 128.68, 127.53, 71.60, 22.17, 21.32. IR (ATIR) ν 3028, 2981, 2931, 1731, 1235, and 1010 cm^{-1}. EI-MS $C_{10}H_{11}O_2Cl$ expected 198.04 m/z observed 198.0 m/z.

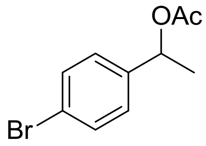

1-(4-Bromophenyl)ethyl acetate (3c) was prepared in an 8 mL microwave vial according to general procedure A using 4-bromoethylbenzene (185 mg, 1 mmol), ammonium iodate (193 mg, 1 mmol), and *N*-hydroxyphthalimide to afford the title complex as a yellow oil (175 mg, 72%). The product was characterized via ^{1}H NMR, ^{13}C NMR, IR, and EI-MS and the spectra obtained matched previously reported spectral values.[8]

^{1}H NMR ($CDCl_3$, 500 MHz, δ) 7.44 (d=8.53 Hz, 2H), 7.23 (d=8.38 Hz, 2H), 5.80 (q, *J* = 6.6 Hz, 1H), 2.04 (s, 3H), 1.50 (d, *J* = 6.8 Hz, 3H). ^{13}C NMR ($CDCl_3$, 126 MHz, δ)

170.22, 140.73, 131.63, 127.86, 121.74, 71.64, 22.14, 21.31. IR (ATIR) v 2955, 2922, 2854, 1739, 1234, 1011 cm^{-1}. EI-MS $C_{10}H_{11}O_2Br$ expected 241.99 m/z observed 242.1 m/z.

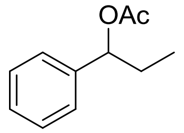

1-Phenylpropyl acetate (3e) was prepared in an 4 mL conical vial according to general procedure B using *n*-propyl benzene (240 mg, 2 mmol), sodium iodate (409.4 mg, 2 mmol), and *N*-hydroxyphthalimide (32 mg, 0.2 mmol) to afford the title complex as a yellow oil (142 mg, 37%). The product was characterized via ^{1}H NMR, ^{13}C NMR, IR and EI-MS and matched spectral data previously reported.[9]

^{1}H NMR (300 MHz, $CDCl_3$) δ 7.31-7.18 (m, 5H), 5.60 (t, *J*=6.96 Hz, 1 H), 2.01 (s, 3 H), 1.91-1.67 (m, 2H), 0.81 (t, *J*= 7.45 Hz, 3H) ^{13}C NMR (75 MHz, $CDCl_3$) δ 170.42, 140.50, 128.34, 127.78, 126.53, 77.32, 29.26, 21.24, 9.89. IR (ATIR) v 3033, 2969, 2935, 2878, 1731, 1233, 1208 cm^{-1}. EI-MS $C_{11}H_{14}O_2$ expected 178.22 m/z observed 178 m/z.

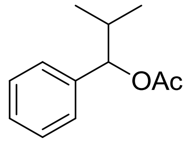

2-Methyl-1-phenylpropyl acetate (3f) was prepared in a 4 mL conical vial according to the general procedure B using isobutylbenzene (0.315 mL, 2 mmol), sodium iodate (409.4 mg, 2 mmol), and *N*-hydroxyphthalimide to afford the title complex as a clear yellow oil (180 mg, 46%). The product was characterized via ^{1}H NMR, ^{13}C NMR, IR, and EI-MS and the spectra obtained matched previously reported spectral values.[17]

^{1}H NMR ($CDCl_3$, 300 MHz) δ 7.31 – 7.24 (m, 5H), 5.43 (d, J = 7.6 Hz, 1H), 2.16 – 1.94 (m, 4 H), 0.94 (d, J = 6.7 Hz, 1H), 0.77 (d, J = 6.8 Hz, 1H). ^{13}C NMR (75 MHz, $CDCl_3$) δ 170.36, 128.16, 127.68, 127.04, 80.96, 33.51, 21.20, 18.71, 18.52. IR (ATIR): 3032.89, 2963.68, 2931.82, 2875.34, 1733.29, 1232.08, 1020.22. EI-MS $C_{12}H_{16}O_2$ expected 192.25 m/z observed 193 m/z.

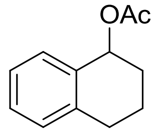

1,2,3,4-Tetrahydronapthalen-1-yl acetate (3g) was prepared in a 4 mL conical vial according to general procedure B at 60 °C using 1,2,3,4-tetrahydronapthalene (288 mg, 2 mmol) sodium iodate (409.4 mg, 2 mmol), and *N*-hydroxyphthalimide (32 mg, 0.2 mmol) to afford the title complex as a clear oil (94.5 mg, 23%). The product was characterized via ^{1}H NMR, ^{13}C NMR, IR, and EI-MS and matched spectral data previously reported.[9]

^{1}H NMR (300 MHz, chloroform-*d*) δ 7.33-7.28 (m, 4 H), 6.03 (br t, *J*= 4.1 Hz, 1 H) 2.98-2.70 (m, 2H), 2.11 (s, 3H), 2.07-1.96 (m, 3H), 1.86-1.80 (m, 1 H). ^{13}C NMR (75 MHz, $CDCl_3$) δ 170.80, 137.93, 134.51, 129.44, 129.08, 128.09, 126.07, 69.96, 29.05, 28.96, 21.50, 18.77. IR (ATIR) v 3024.64, 2939.76, 2869.08, 1728.35, 1233.26, 1210.73 cm^{-1}. EI- MS $C_{12}H_{14}O_2$ expected 190.24 m/z observed 190 m/z.

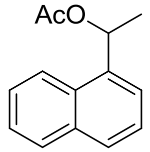

1-(Naphthalen-1-yl)ethyl acetate (3h) was prepared in an 8 mL microwave vial according to general procedure A using 1-ethylnapthylene (156 mg, 1 mmol), ammonium iodate (193 mg, 1 mmol), and *N*-hydroxyphthalimide to afford the title complex as a yellow oil (75 mg, 35%). The product was characterized via ^{1}H NMR, ^{13}C NMR, IR and EI-MS and the spectra obtained matched previously reported spectral values.[11]

^{1}H NMR ($CDCl_3$, 500 MHz,) δ 7.99 (d, *J* = 8.5 Hz, 1H), 7.77 (d=7.97 Hz, 1H), 7.69 (d, *J* = 8.18 Hz, 1H), 7.51 (d, *J* = 7.33 Hz, 1H), 7.46 – 7.41 (m, 1H), 7.41 – 7.33 (m, 2H), 6.56 (q, *J* = 6.6 Hz, 1H), 2.02 (s, 3H), 1.61 (d, *J* = 6.6 Hz, 3H). ^{13}C NMR ($CDCl_3$, 126 MHz, δ) 170.39, 137.40, 133.81, 130.24, 128.92, 128.45, 126.31, 125.68, 125.36, 123.19, 123.16, 69.45, 21.71, 21.41. (ATIR) v 3051, 2982, 2933, 1733, 1369, 1231 cm^{-1}. EI-MS $C_{14}H_{14}O_2$ expected 214.10 m/z observed 214.2 m/z.

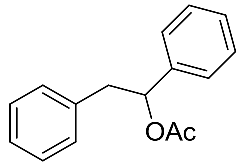

1,2-diphenyl acetate (3i) was prepared in an 8 mL microwave vial according to the general procedure A using bibenzyl (182 mg, 1 mmol), ammonium iodate (193 mg, 1 mmol), and *N*-hydroxyphthalimide to afford the title complex as a yellow oil (127 mg, 52%). The product was characterized via ^{1}H NMR, ^{13}C NMR, IR, and EI-MS and the spectra obtained matched previously reported spectral values.[15]

^{1}H NMR ($CDCl_3$, 500 MHz, δ) δ 7.24 – 7.07 (m, 8H), 7.03-6.99 (m, 2H), 5.86 (dd, *J* = 8.0, 6.0 Hz, 1H), 3.10 (dd, *J* = 13.8, 8.0 Hz, 1H), 2.96 (dd, *J* = 13.8, 6.1 Hz, 1H), 1.91 (s, 3H). ^{13}C NMR ($CDCl_3$, 126 MHz, δ) 169.06, 138.99, 135.96, 128.46, 127.31, 127.18, 126.92, 125.56, 125.50, 75.58, 41.93, 20.15. IR (ATIR) v 3063, 3031, 2947, 1734, 1230, 1020, 696 cm^{-1}. EI-MS $C_{14}H_{12}$ (desaturation) expected 180.09 m/z observed 180.2 m/z.

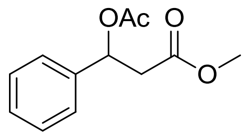

Methyl 3-acetoxy-3-phenylpropanoate (3j) was prepared in an 4 mL conical vial according to the general procedure B using methyl-3-phenylpropanoate (328 mg, 2 mmol), sodium iodate (409.4 mg, 2 mmol), and *N*-hydroxyphthalimide to afford the title complex as a clear yellow oil (111 mg, 25%). The product was characterized via ^{1}H NMR, ^{13}C NMR, IR, and EI-MS and the spectra obtained matched previously reported spectral values.[12]

^{1}H NMR ($CDCl_3$, 300 MHz) δ 7.32-7.08 (m, 5H), 6.09 (dd, *J*=8.99, 5.15 Hz, 1H), 3.58 (s, 3H), 2.90 (dd, *J*= 15.92, 9.01 Hz, 1H), 2.68 (dd, *J*=15.68, 5.18 Hz, 1H), 1.96 (s, 3H). ^{13}C NMR (75 MHz, $CDCl_3$) δ 170.21, 169.75, 139.28, 128.63, 128.39, 126.46, 72.07, 51.83, 41.19, 20.96 IR (ATIR) v 3032.08, 2931.06, 1737.90, 1226.52, 1184.94, 1020.03 cm^{-1}. EI-MS $C_{12}H_{14}O_4$ expected 222.23 m/z observed 222 m/z.

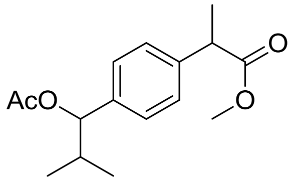

Methyl 2-methyl-(4-(1-acetoxy-2-methylpropyl)phenyl)propanoate (3k) was prepared in an 8 mL microwave vial according to general procedure A using ibuprofen methyl ester (220 mg, 1 mmol), ammonium iodate (193 mg, 1 mmol), and *N*-hydroxyphthalimide to afford the title complex as a clear oil (216 mg, 78%)

^{1}H NMR ($CDCl_3$, 500 MHz, δ) 7.30 – 7.22 (m, 4H), 5.47 (d, *J* = 7.6 Hz, 1H), 3.73 (q, J=6.92 Hz, 1H), 3.68 (s, 3H), 2.13-2.05 (m, 4H), 1.51 (dd, *J* = 7.2, 1.1 Hz, 3H), 0.98 (d, *J* = 6.7 Hz, 3H), 0.81 (d, *J* = 6.8 Hz, 3H). ^{13}C NMR (126 MHz, $CDCl_3$ δ) 174.99, 174.94, 170.41, 139.84, 139.82, 138.63, 127.33, 127.28, 80.66, 52.06, 45.11, 45.09, 33.50, 21.22, 18.77, 18.62, 18.57, 18.48. IR (ATIR) v 2966, 2875, 1732, 1371, 1234, 1207, 1018 cm^{-1}. HR ESI-MS expected $C_{16}H_{22}O_4Na$ $[M+Na]^+$ 301.1416 m/z observed 301.1255 m/z.

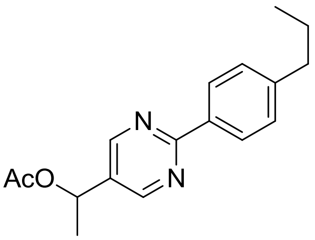

1-(2-(4-Propylphenyl)pyrimidin-5-yl)ethyl acetate (3l) was prepared in an 8 mL microwave vial according to general procedure A using 5-ethyl-2-(4-propylphenyl)pyrimidine (226 mg, 1 mmol), ammonium iodate (193 mg, 1 mmol), and *N*-hydroxyphthalimide to afford the title complex as a yellow solid (217 mg, 76%)

^{1}H NMR ($CDCl_3$, 500 MHz, δ) 8.69 (s, 2H), 8.37 – 8.33 (m, 2H), 7.50 – 7.47 (m, 2H), 5.96 (q, *J* = 6.6 Hz, 1H), 2.63 (t, J=7.85 Hz, 2H), 2.12 (s, 3H), 1.71 (h, *J* = 7.4 Hz, 2H), 1.58 (d, *J* = 6.6 Hz, 3H), 1.01 (t, *J* = 7.3 Hz, 3H). ^{13}C NMR ($CDCl_3$, 500 MHz, δ) 170.46, 162.20, 157.13, 143.99, 136.85, 133.10, 128.19, 126.32, 72.09, 32.17, 23.94, 22.21, 21.38, 13.61. IR (ATIR) v 2960, 2931, 2872, 1733, 1428, 1233 cm^{-1}. HR-ESI-MS $C_{17}H_{21}N_2O_2$, $[M+H]^+$ expected 285.1603 m/z observed 285.1565 m/z.

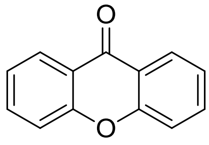

9*H*-Xanthen-9-one (5a) was prepared in an 8 mL microwave vial according to general procedure A using xanthene (182 mg, 1 mmol), ammonium iodate (193 mg, 1 mmol), and *N*-hydroxyphthalimide to afford the title complex as a white solid (112 mg, 57%). The product was characterized via ^{1}H NMR, ^{13}C NMR, IR, and EI-MS and the spectra obtained matched previously reported spectral values.[16]

^{1}H NMR ($CDCl_3$, 300 MHz, δ) 8.36 (dd, *J* = 8.0, 1.8 Hz, 2H), 7.75 (ddd, *J* = 8.6, 7.0, 1.8 Hz, 2H), 7.51 (dd, *J* = 8.3, 1.2 Hz, 2H), 7.40 (ddd, *J* = 8.1, 7.0, 1.1 Hz, 2H). ^{13}C NMR ($CDCl_3$, 75 MHz, δ) 176.18, 155.00, 133.76, 125.58, 122.81, 120.62, 116.87. IR (ATIR) v 3080, 3032, 1654, 1605, 1476, 1455, 1330, 755 cm^{-1}. EI-MS $C_{13}H_8O_2$ expected 196.05 m/z observed 196.0 m/z.

A mixture of **fluoren-9-acetate** and **fluorenone (5b and 5c)** was prepared in a 5 mL conical vial according to general procedure B using fluorine (332 mg, 2 mmol), sodium

iodate (409.4 mg, 2 mmol), and *N*-hydroxyphthalimide to afford a mixture of the title complexes (1.5:1 acetate to ketone) as a clear yellow oil (211 mg). These compounds were unable to be separated via flash column chromatography and were characterized as a mixture of compounds (Figures S5-S10).

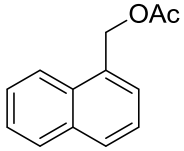

Naphthalene-1-ylmethyl acetate (7a) was prepared in an 8 mL microwave vial according to general procedure A using 1-methyl naphthalene (142 mg, 1 mmol), ammonium iodate (193 mg, 1 mmol), and *N*-hydroxyphthalimide to afford the title complex as a yellow oil (51 mg, 25%). The product was characterized via ^{1}H NMR, ^{13}C NMR and EI-MS and the spectra obtained matched previously reported spectral values.[13-14]

^{1}H NMR ($CDCl_3$, 300 MHz, δ) δ 8.03 (d, J = 8.35 Hz, 1H), 7.86 (m, 2H), 7.58 – 7.40 (m, 4H), 5.58 (s, 2H), 2.09 (s, 3H). ^{13}C NMR ($CDCl_3$, 75 MHz, δ) 171.01, 133.76, 131.65, 131.46, 129.36, 128.77, 127.55, 126.62, 126.00, 125.33, 123.58, 64.62, 21.06. IR (ATIR) v 3048.11, 2930.50, 1734.37, 1221.30, 1168.59 cm^{-1}. EI-MS $C_{13}H_{11}O_2$ expected 200.08 m/z observed 200.2 m/z.

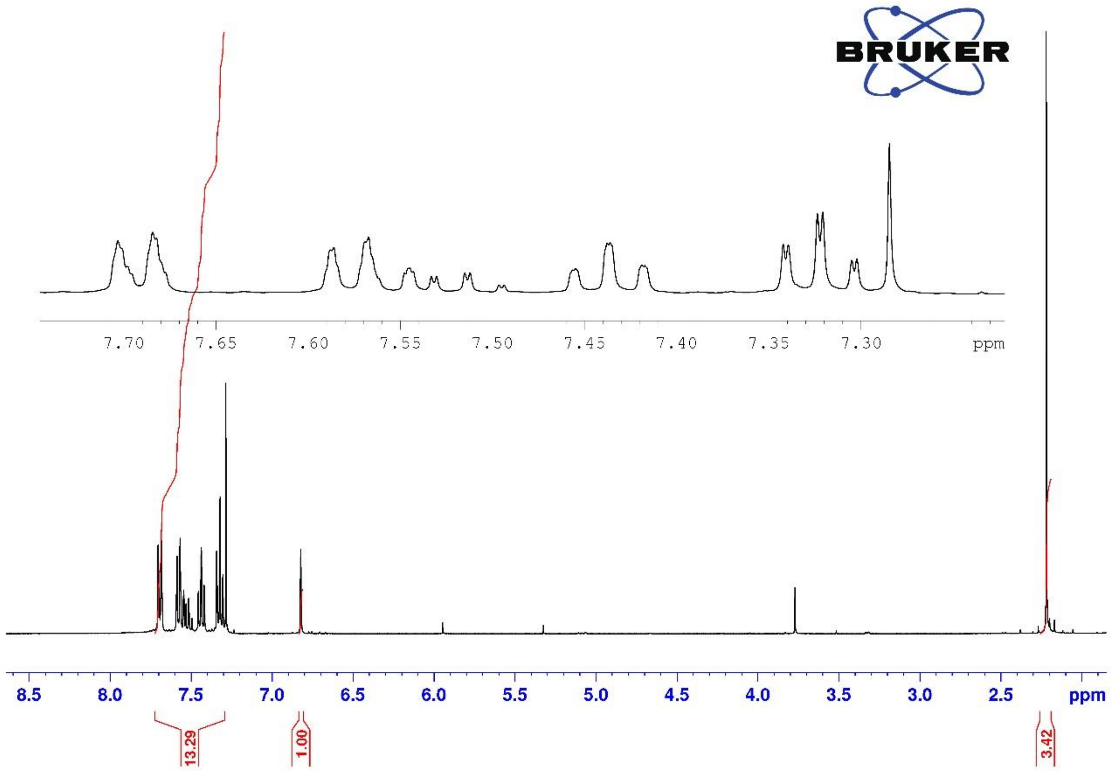

Figure S5. ^{1}H NMR (400 MHz) of the mixture of **fluoren-9-acetate** and **fluorenone** produced via catalytic oxidation**.**

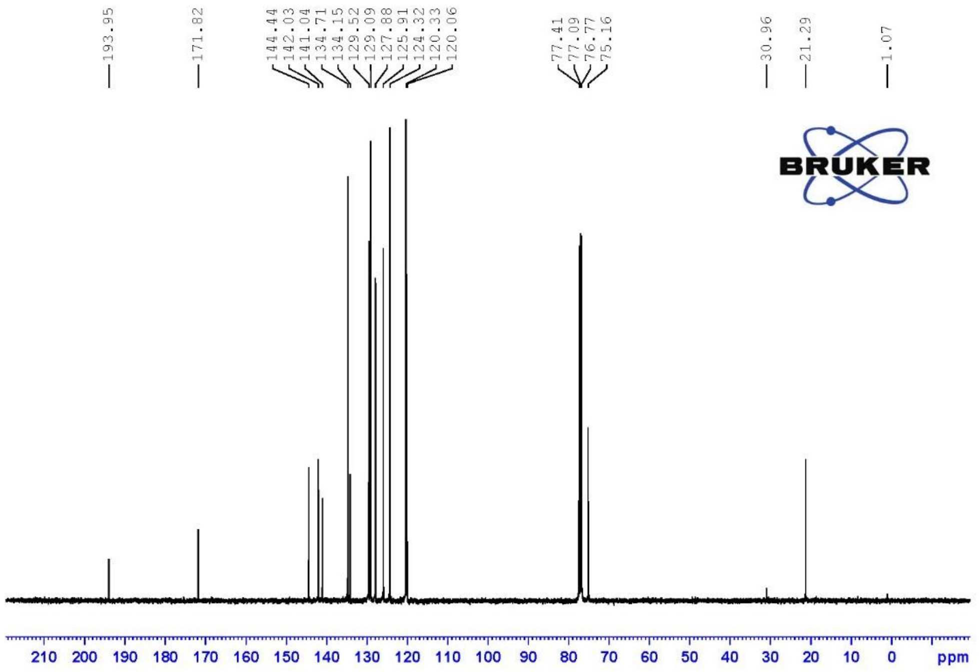

Figure S6. [13] C NMR of the mixture of **fluoren-9-acetate** and **fluorenone** produced via catalytic oxidation**.**

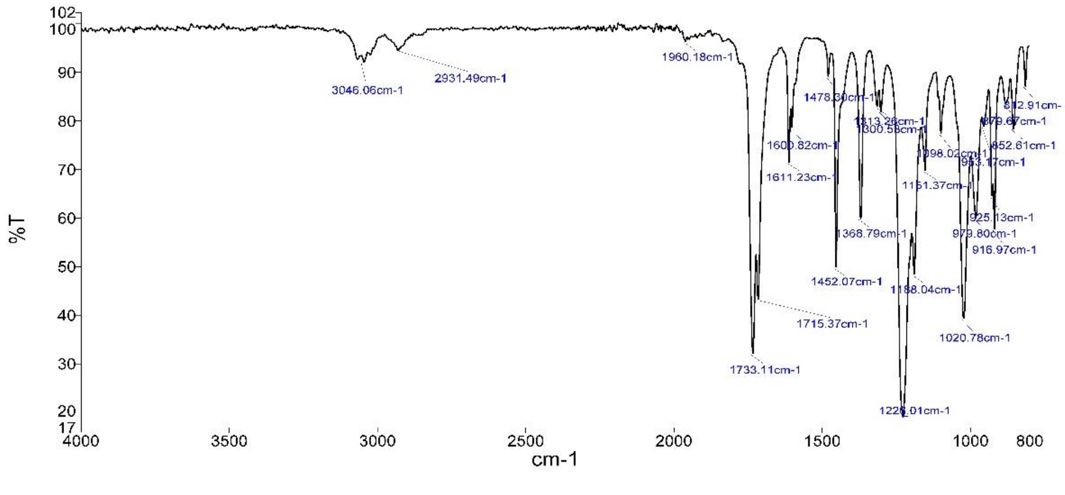

Figure S7. ATIR of the mixture of **fluoren-9-acetate** and **fluorenone**

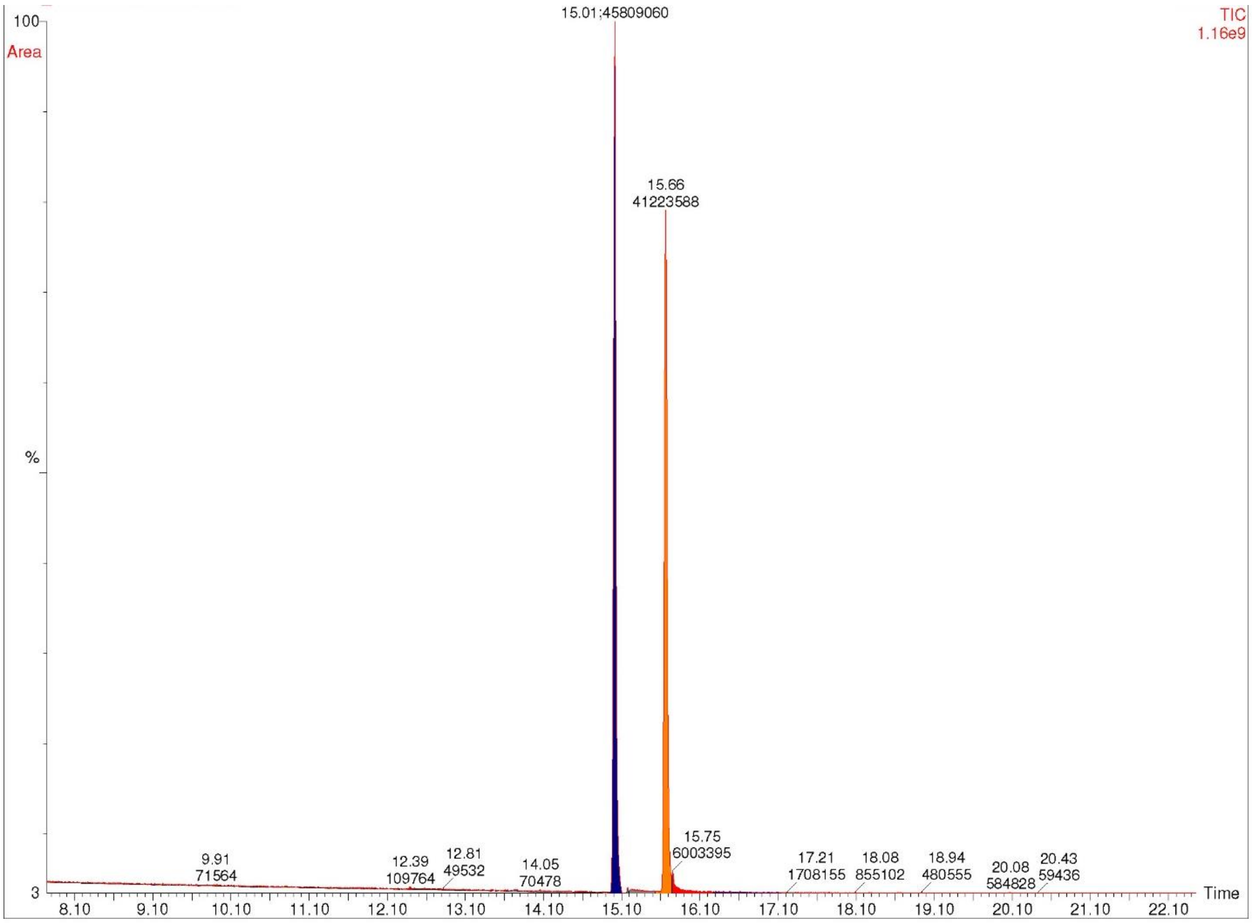

Figure S8. Gas chromatogram of the mixture of **fluoren-9-acetate** and **fluorenone** generated via catalytic oxidation of fluorene.

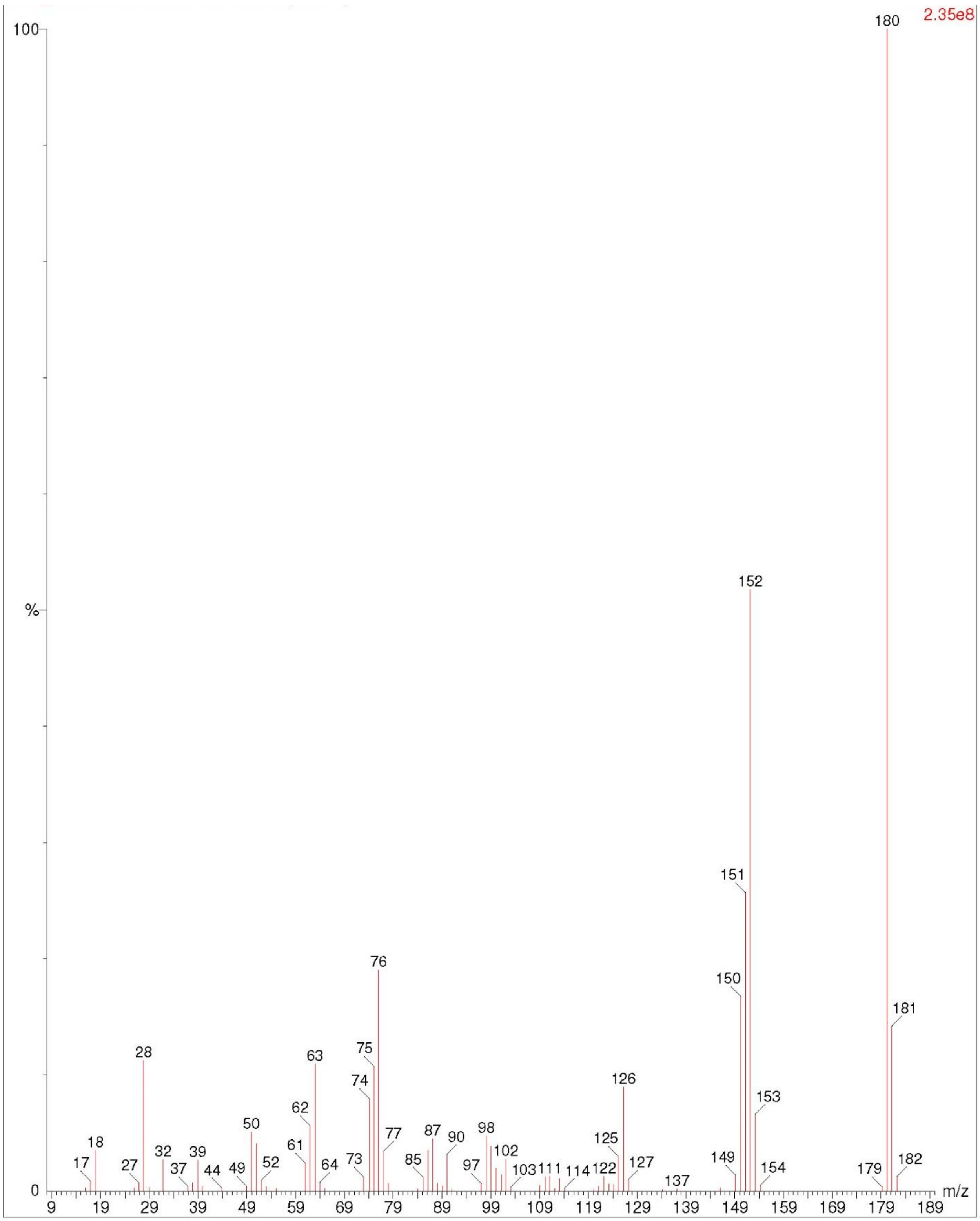

Figure S9. EI-MS for fluorenone (retention time 15.01 minutes in Figure S8).

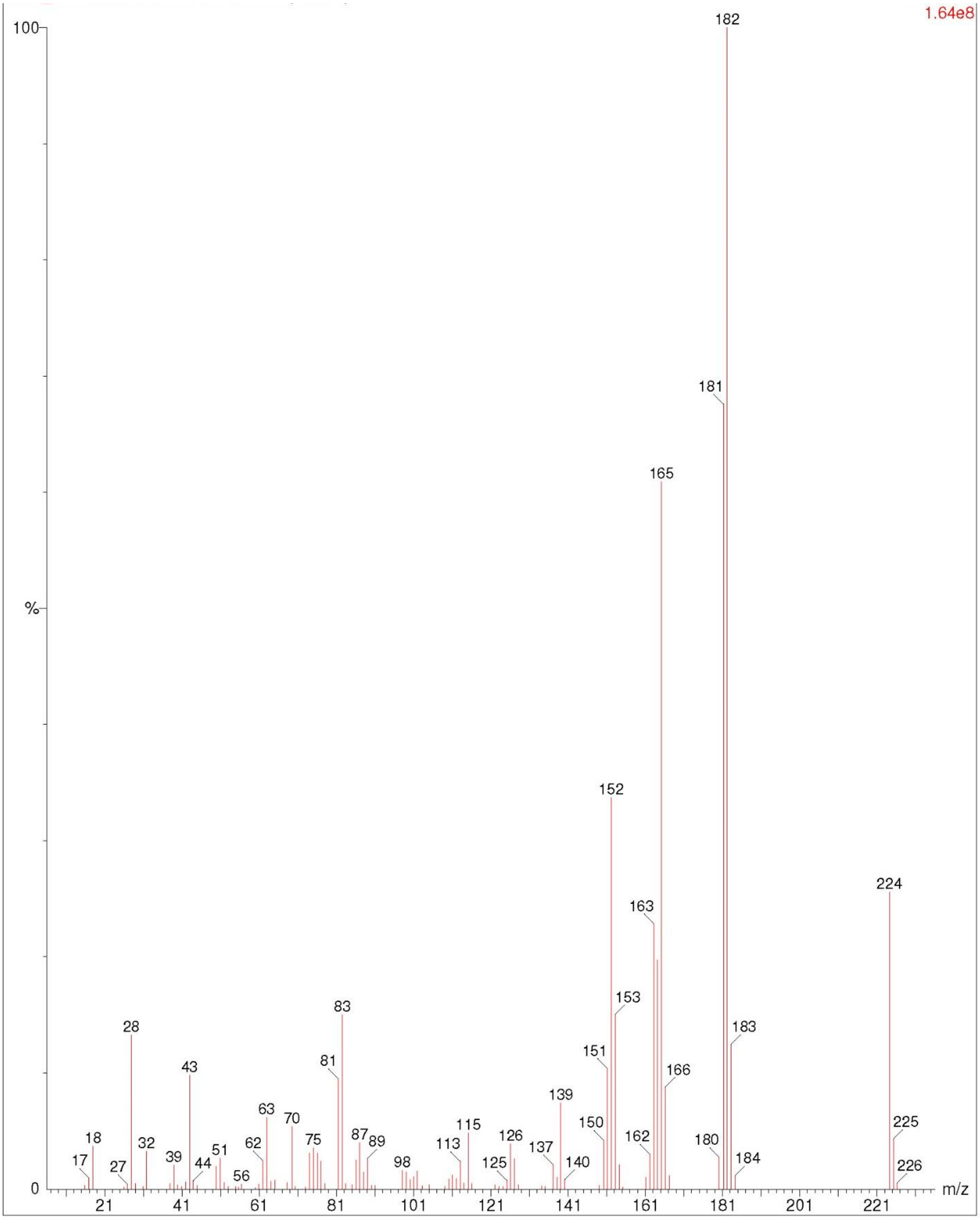

Figure S10. EI-MS for fluorenyl acetate (retention time 15.66 min in Figure S8)

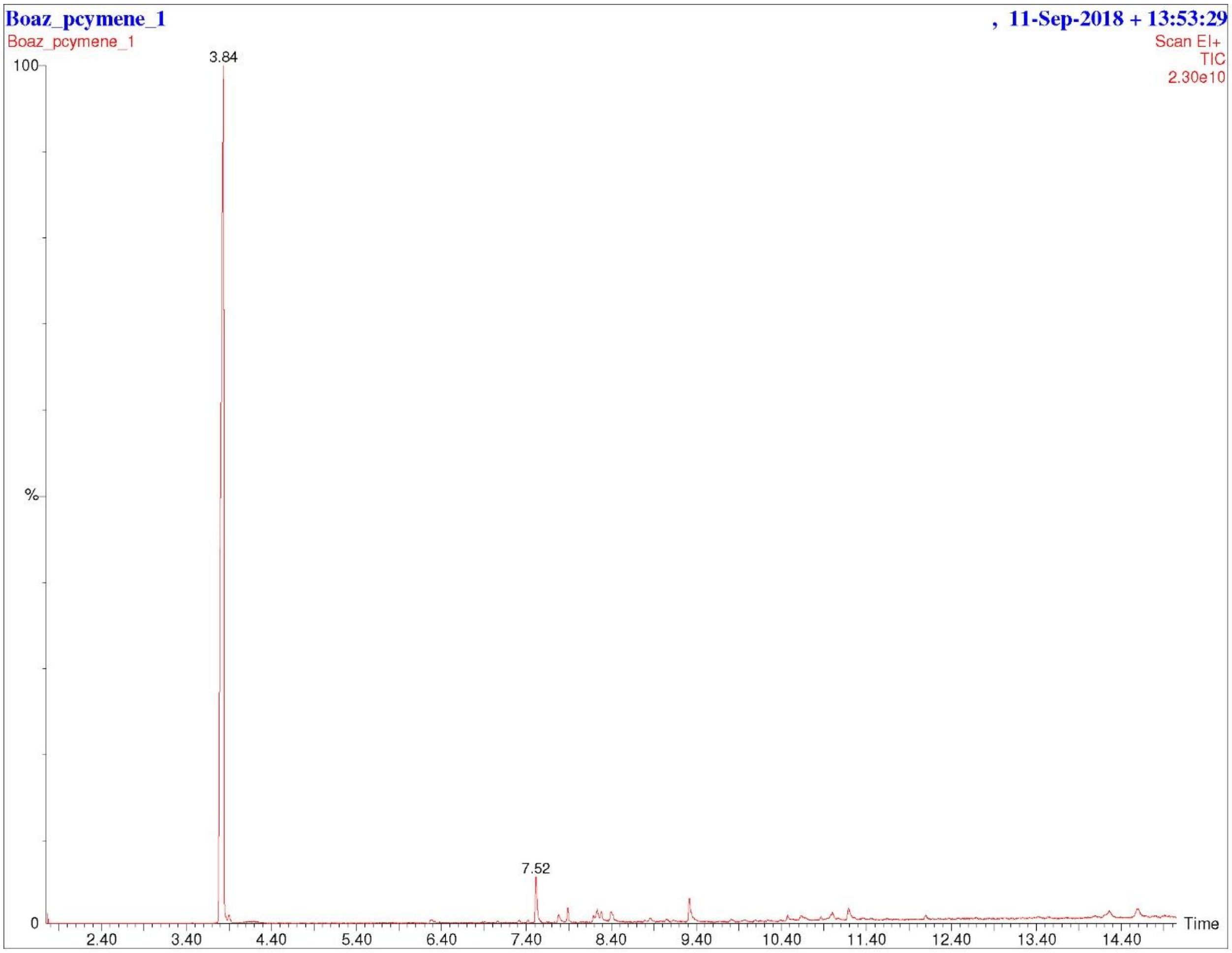

Figure S11. Gas chromatogram of the products of the catalytic oxidation of *p*-cymene. Unreacted starting material is shown at a retention time of 3.8 min. and acetoxylated product is shown at 7.5 min.

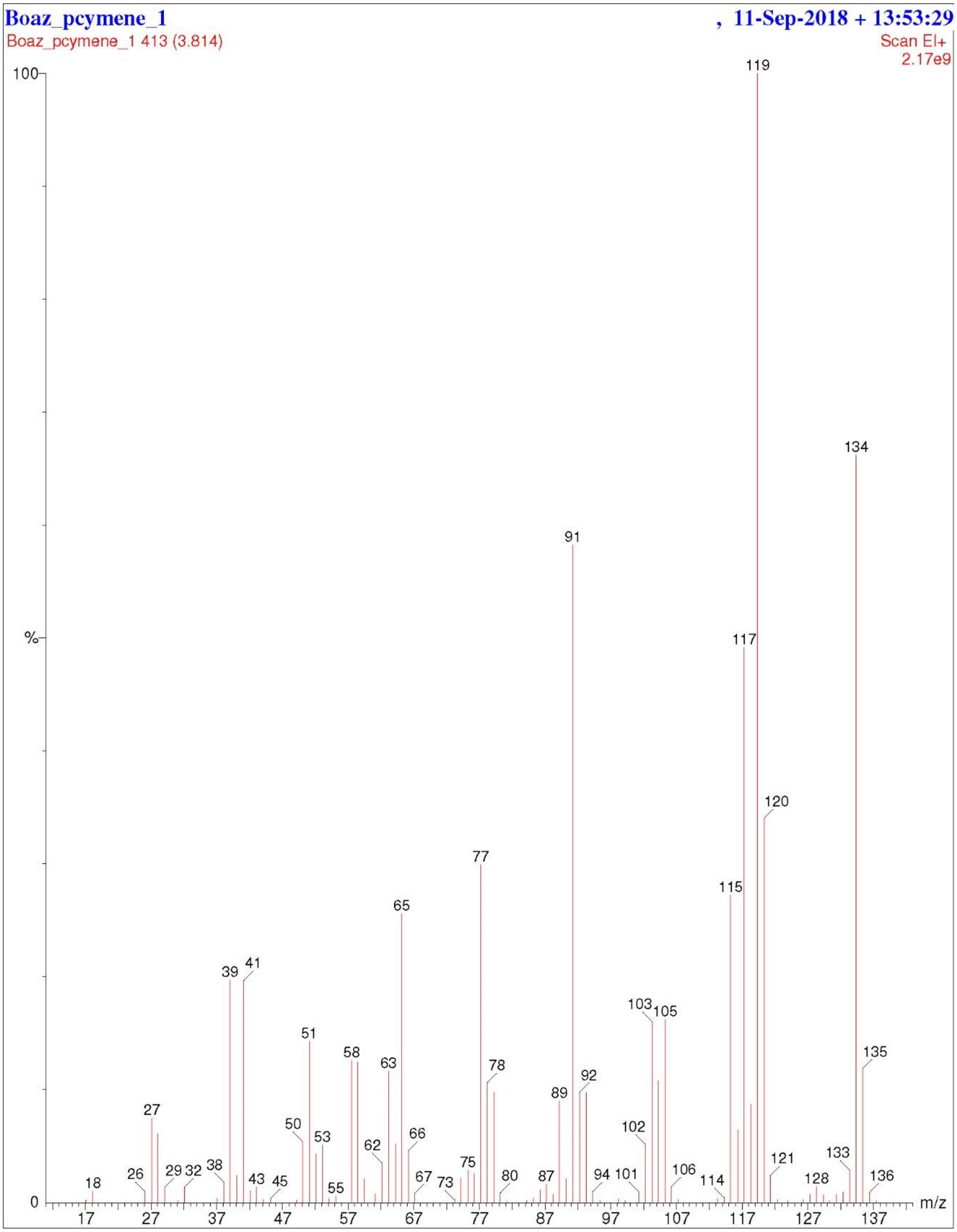

Figure S12. EI-MS for unreacted *p*-cymene (retention time 3.8 min. in Figure S11).

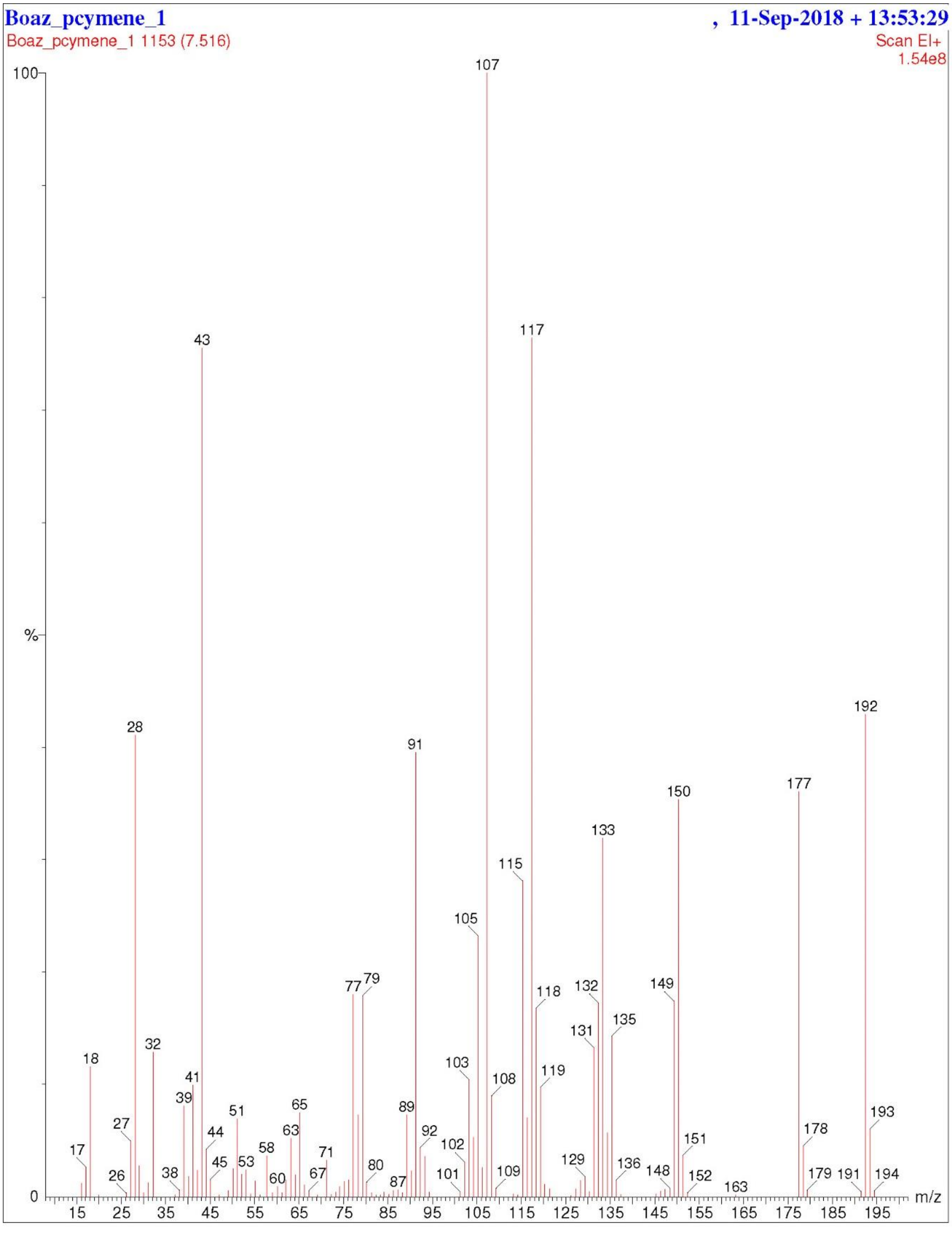

Figure S13. EI-MS for acetoxylated product of *p*-cymene reaction (retention time 7.5 min. in Figure S11).

NMR Spectra for acetate products produced catalytically

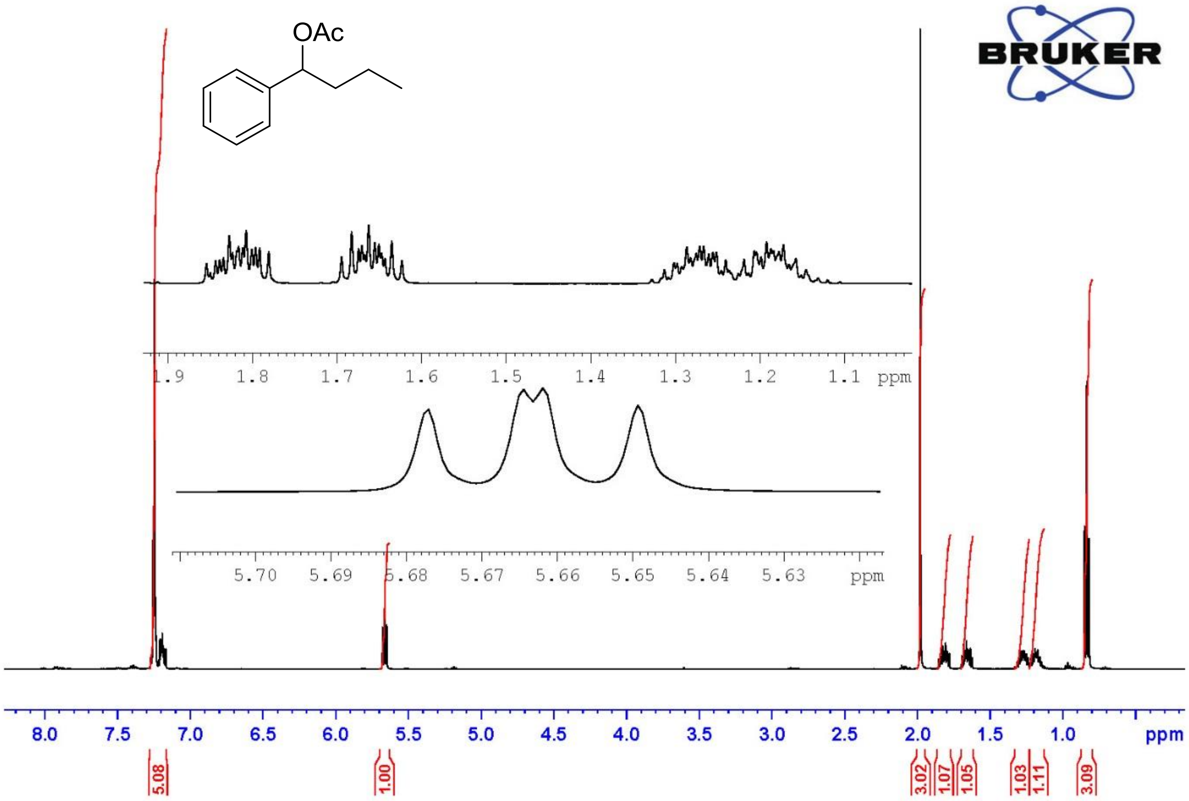

Figure S14. ^{1}H NMR of 1-phenylbutyl acetate (**3a**). Please note that the small number of unlabeled peaks represent a trace amount of an unknown impurity that co-purified with the reported product.

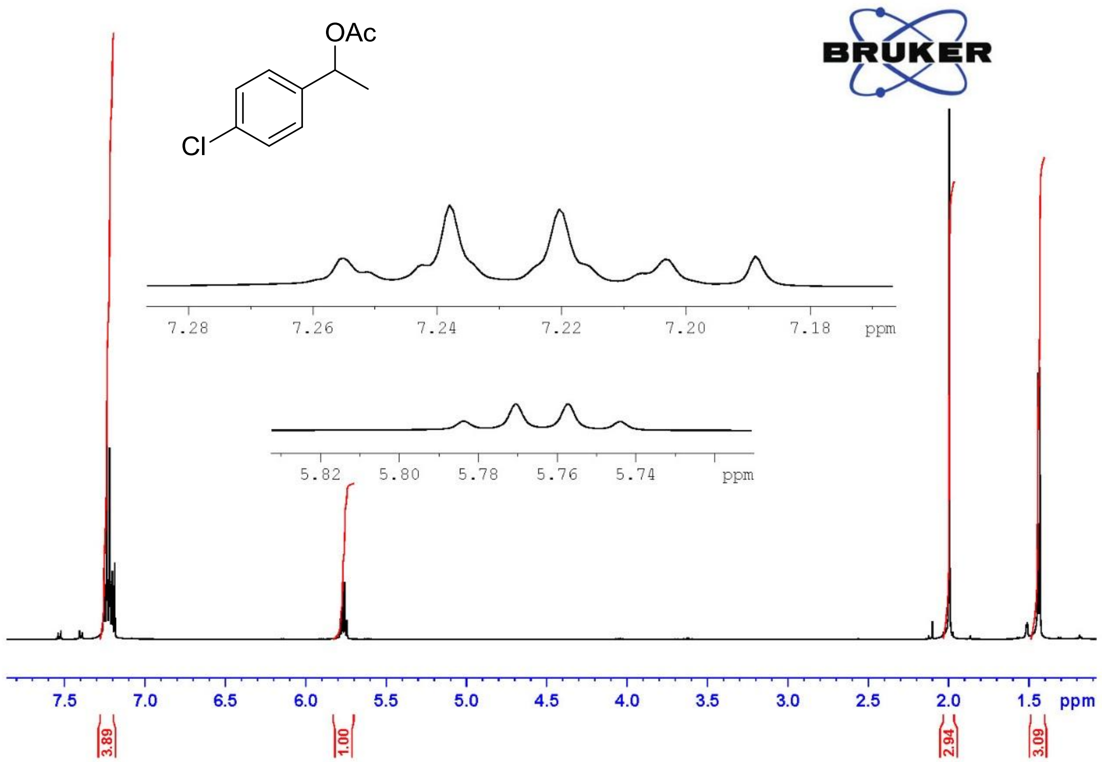

Figure S15. ^{1}H NMR of 1-(4-chlorophenyl)ethyl acetate (**3b**). Please note that the small number of unlabeled peaks represent a trace amount of an unknown impurity that co-purified with the reported product.

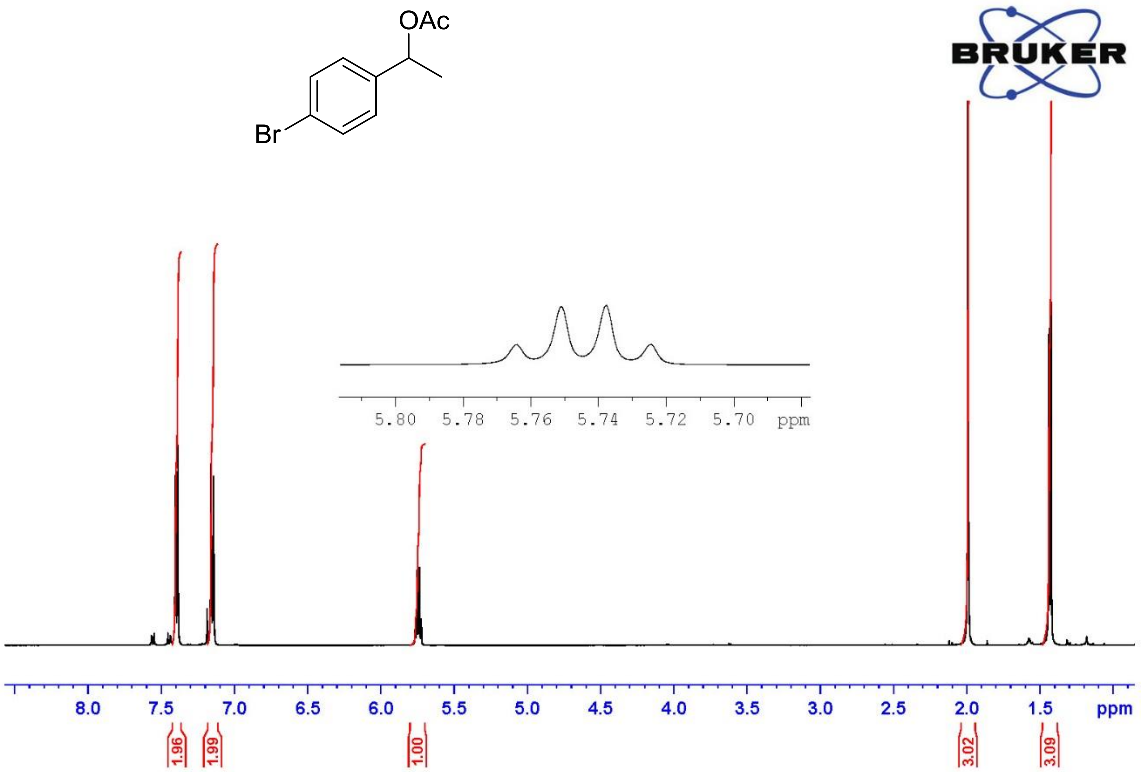

Figure S16. ^{1}H NMR of 1-(4-bromophenyl)ethyl acetate (**3c**). Please note that the small number of unlabeled peaks represent a trace amount of an unknown impurity that co-purified with the reported product.

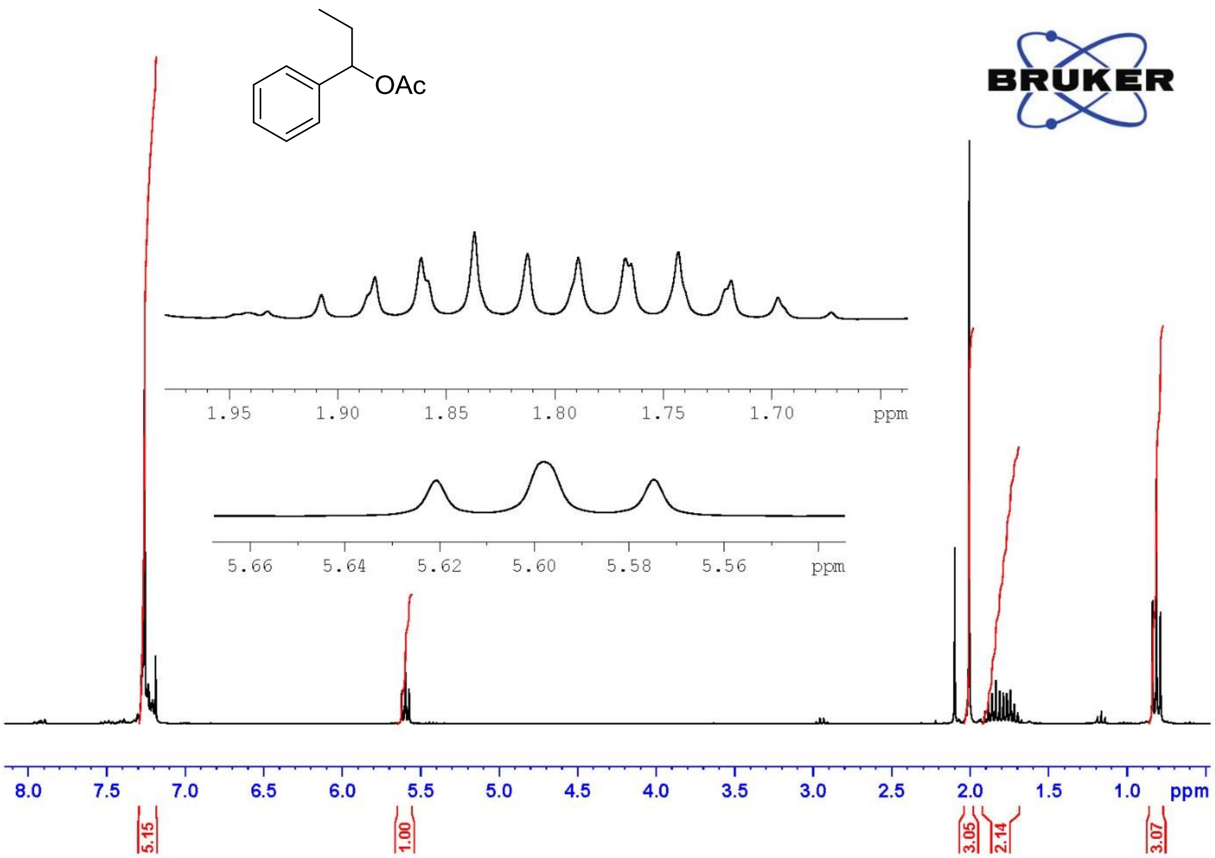

Figure S17. ^{1}H NMR of 1-phenylpropyl acetate (**3e**). Please note that the small number of unlabeled peaks represent a trace amount of an unknown impurity that co-purified with the reported product.

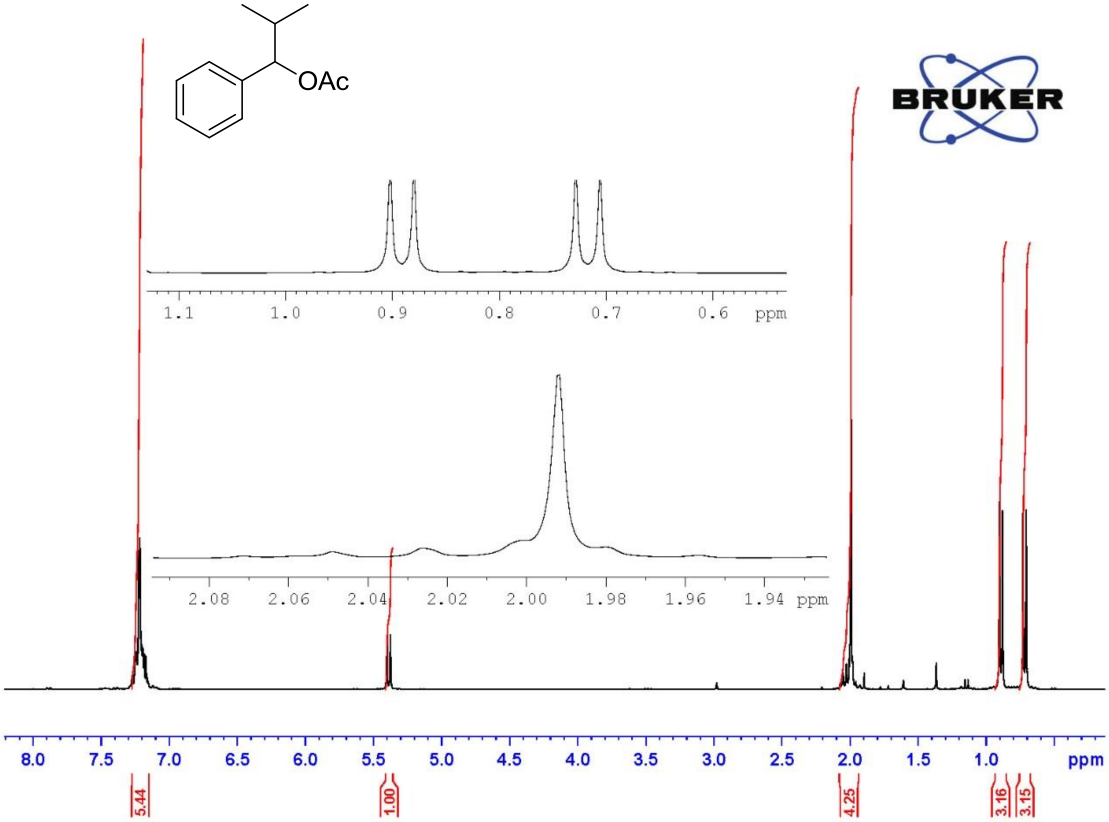

Figure S18. ^{1}H NMR of 2-methyl-1-phenylpropyl acetate (**3f**).

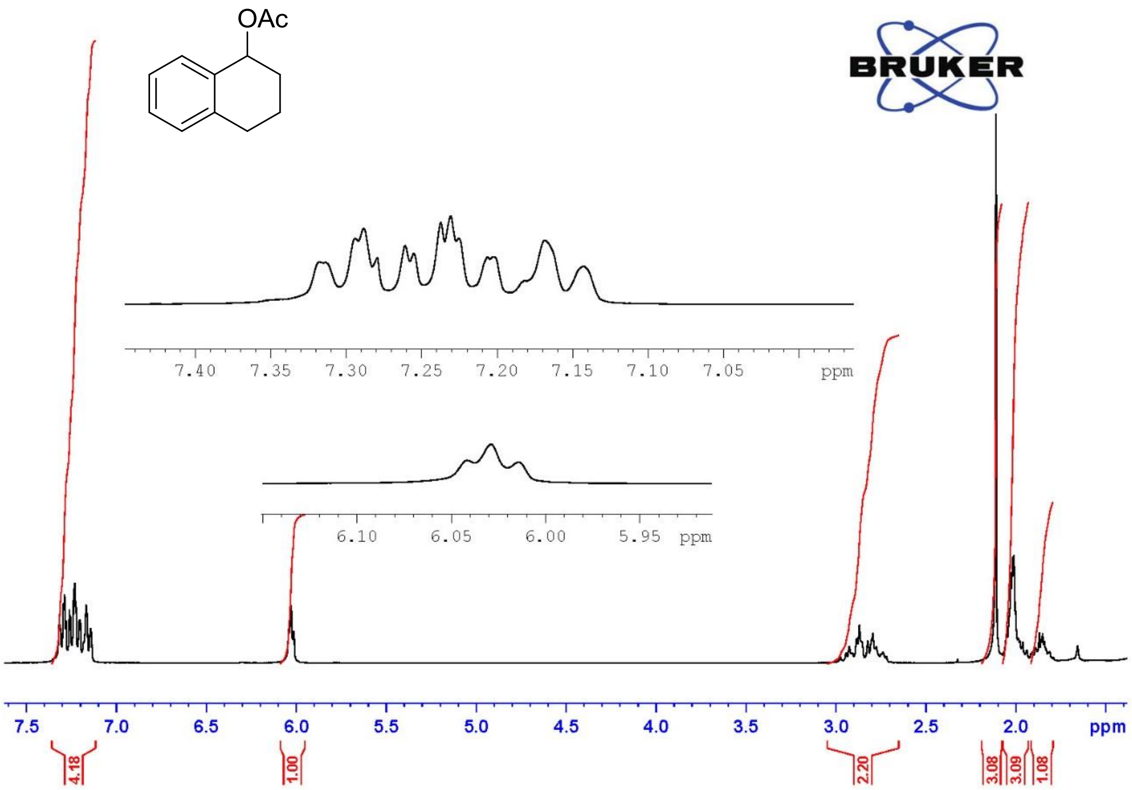

Figure S19. ^{1}H NMR of 1,2,3,4-tetrahydronapthalen-1-yl acetate (**3g**).

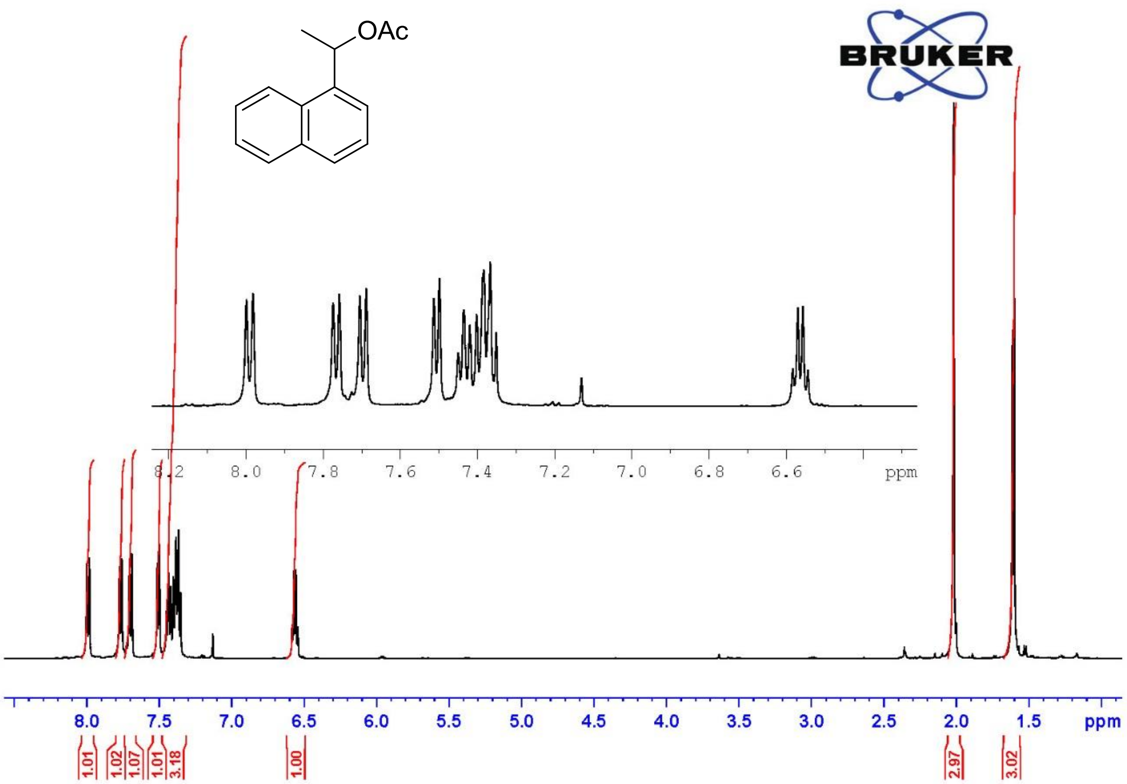

Figure S20. ^{1}H NMR of 1-(naphthalen-1-yl)ethyl acetate (**3h**).

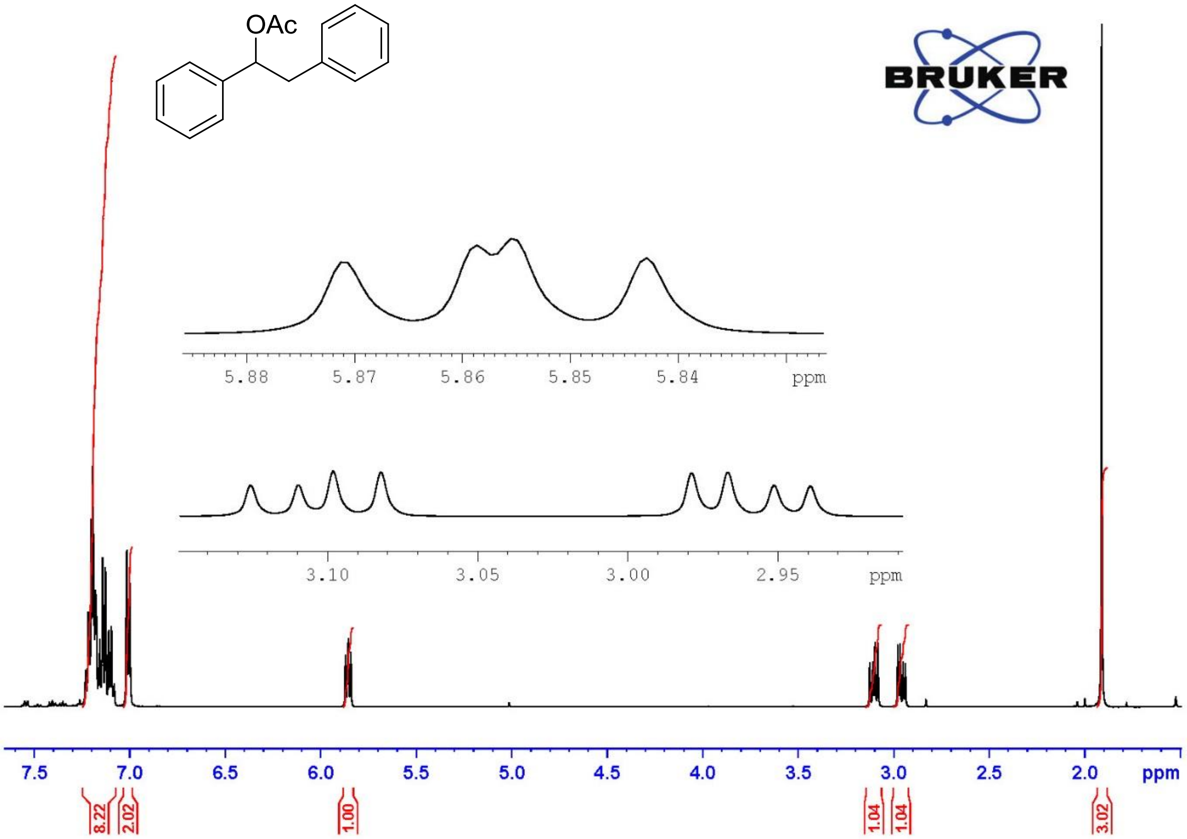

Figure S21. ^{1}H NMR of 1,2-diphenylethyl acetate (**3i**). Please note that the small number of unlabeled peaks represent a trace amount of an unknown impurity that co-purified with the reported product.

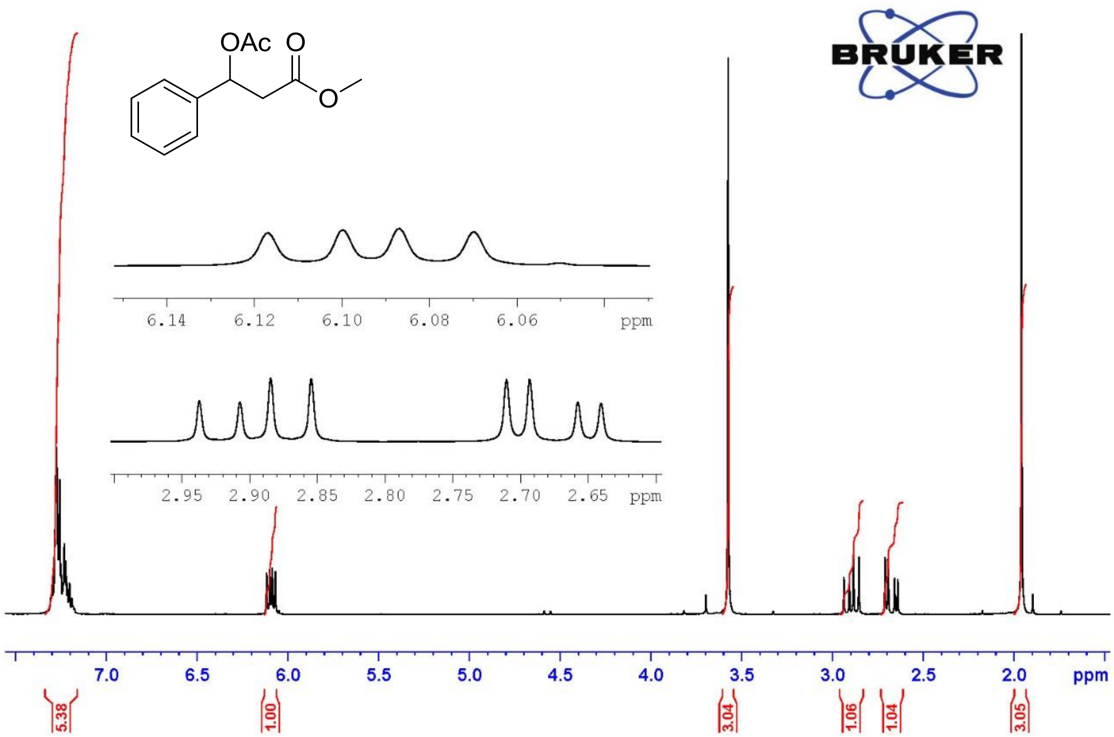

Figure S22. ^{1}H NMR of methyl 3-acetoxy-3-phenylpropanoate (**3j**).

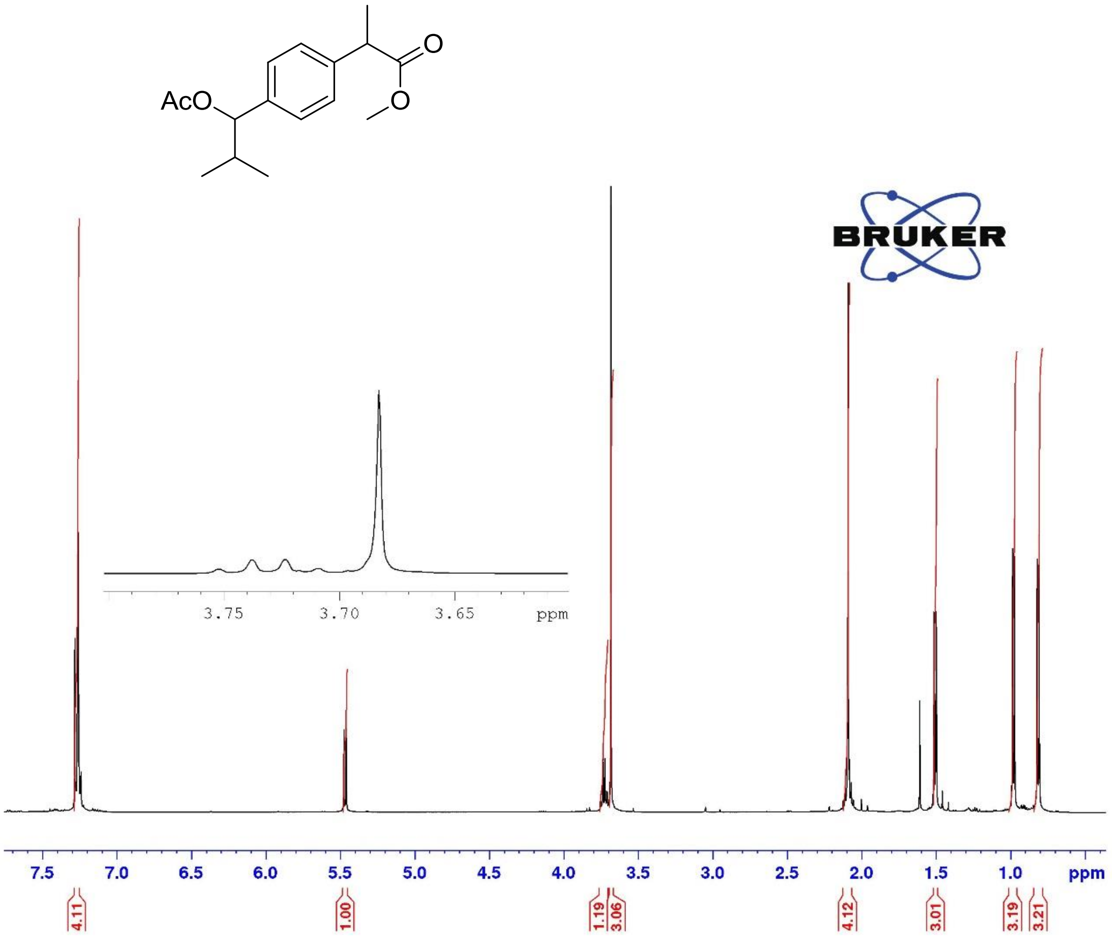

Figure S23. ^{1}H NMR of 2-methyl-(4-(1-acetoxy-2-methylpropyl)phenyl)propanoate (**3k**).

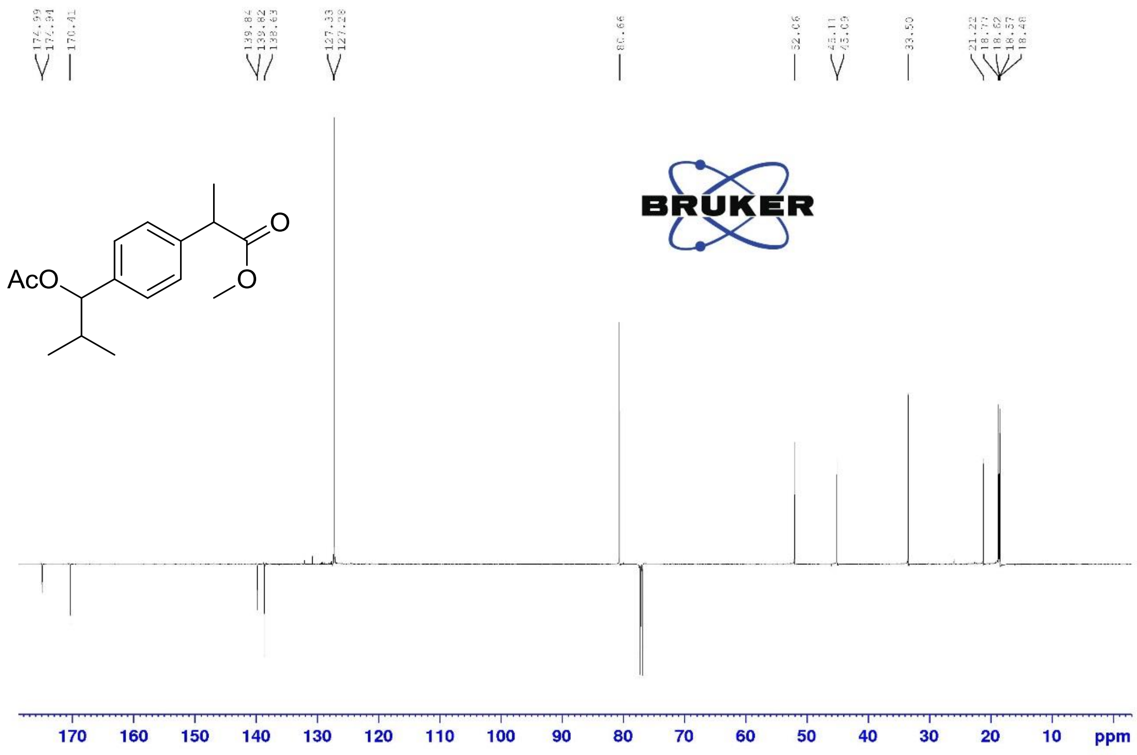

Figure S24. ^{13}C NMR of 2-methyl-(4-(1-acetoxy-2-methylpropyl)phenyl)propanoate (**3k**).

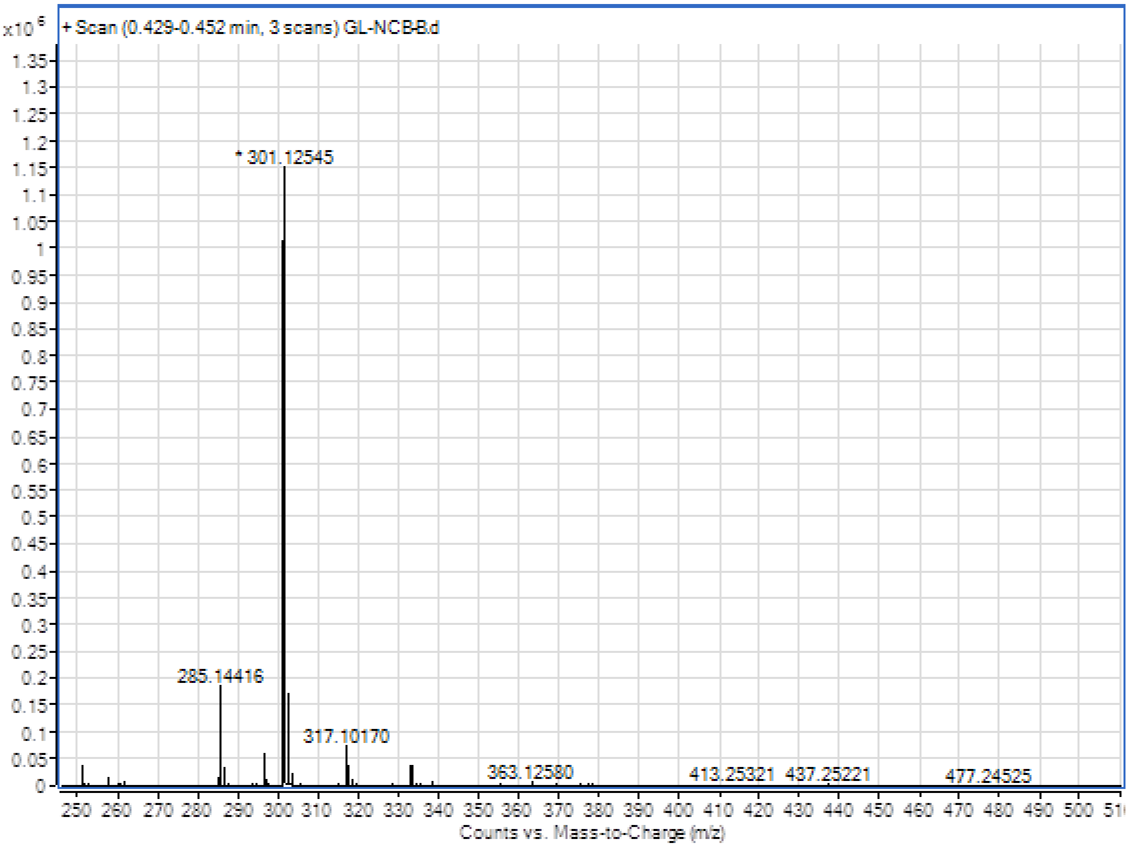

Figure S25. HR-ESI-MS of 2-methyl-(4-(1-acetoxy-2-methylpropyl)phenyl)propanoate (**3k**).

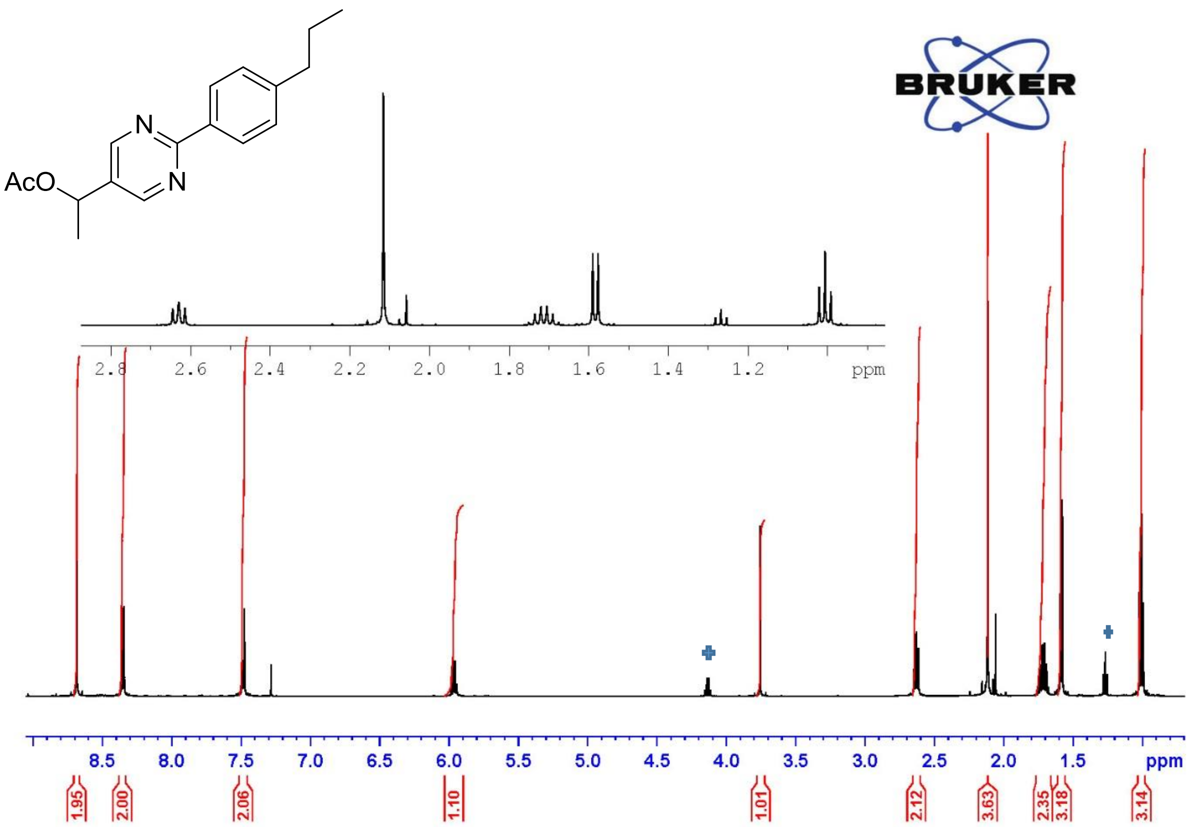

Figure S26. ^1H NMR of 1-(2-(4-propylphenyl)pyrimidin-5-yl)ethyl acetate (**3l**). Please note that + signifies a residual solvent signal.

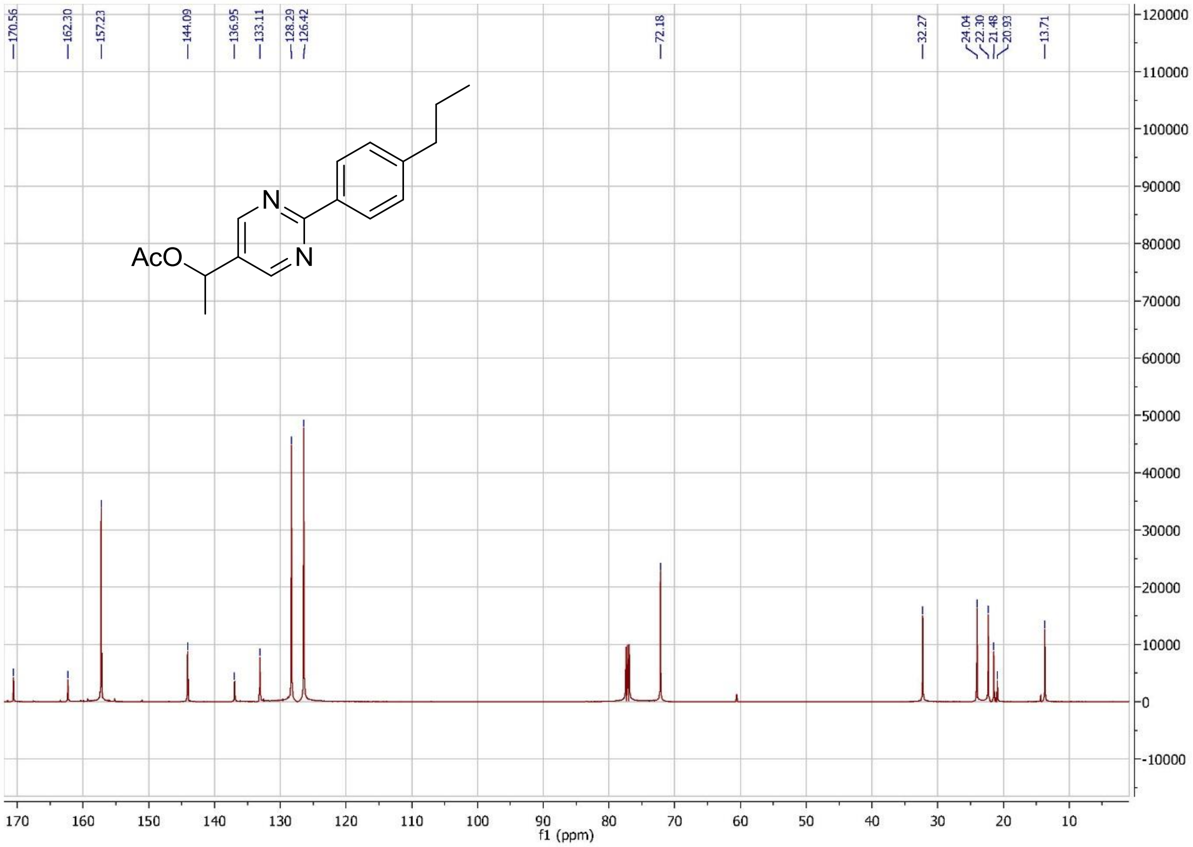

Figure S27. ^{13}C NMR of 1-(2-(4-propylphenyl)pyrimidin-5-yl)ethyl acetate (**3l**).

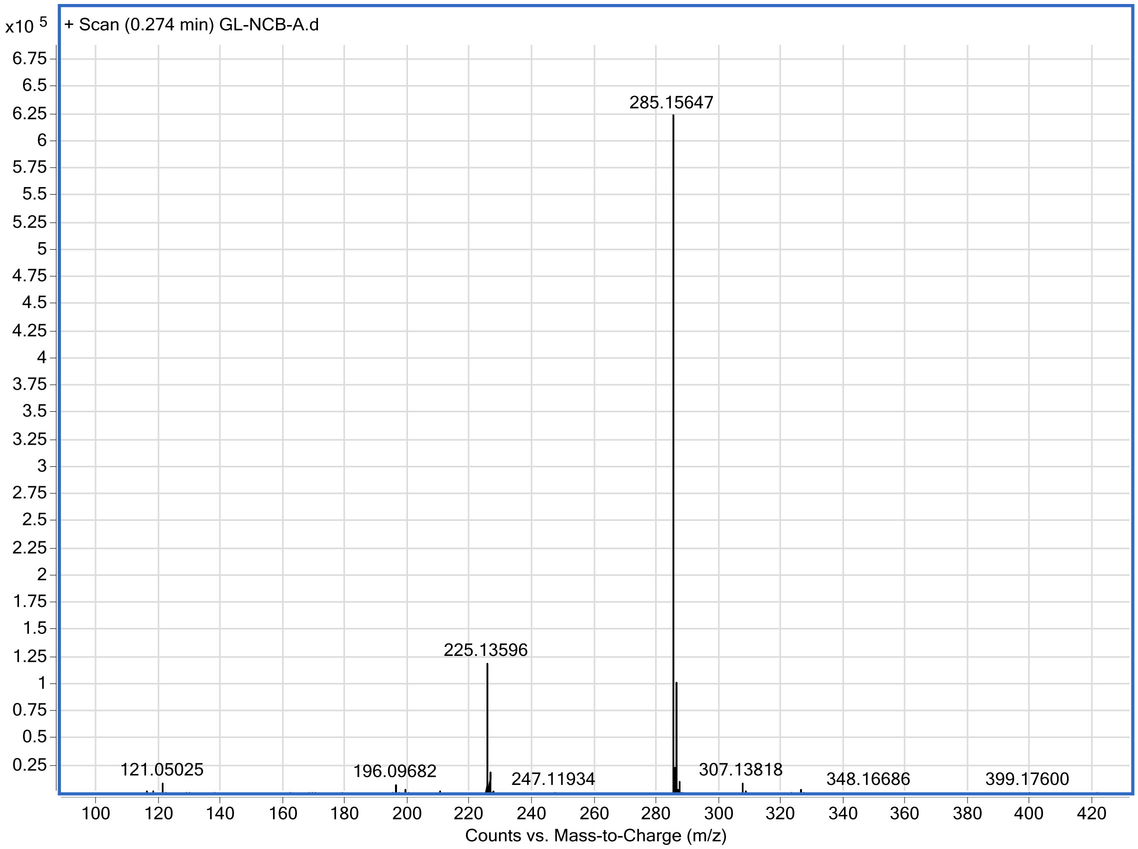

Figure S28. HR-ESI-MS of 1-(2-(4-propylphenyl)pyrimidin-5-yl)ethyl acetate (**3l**).

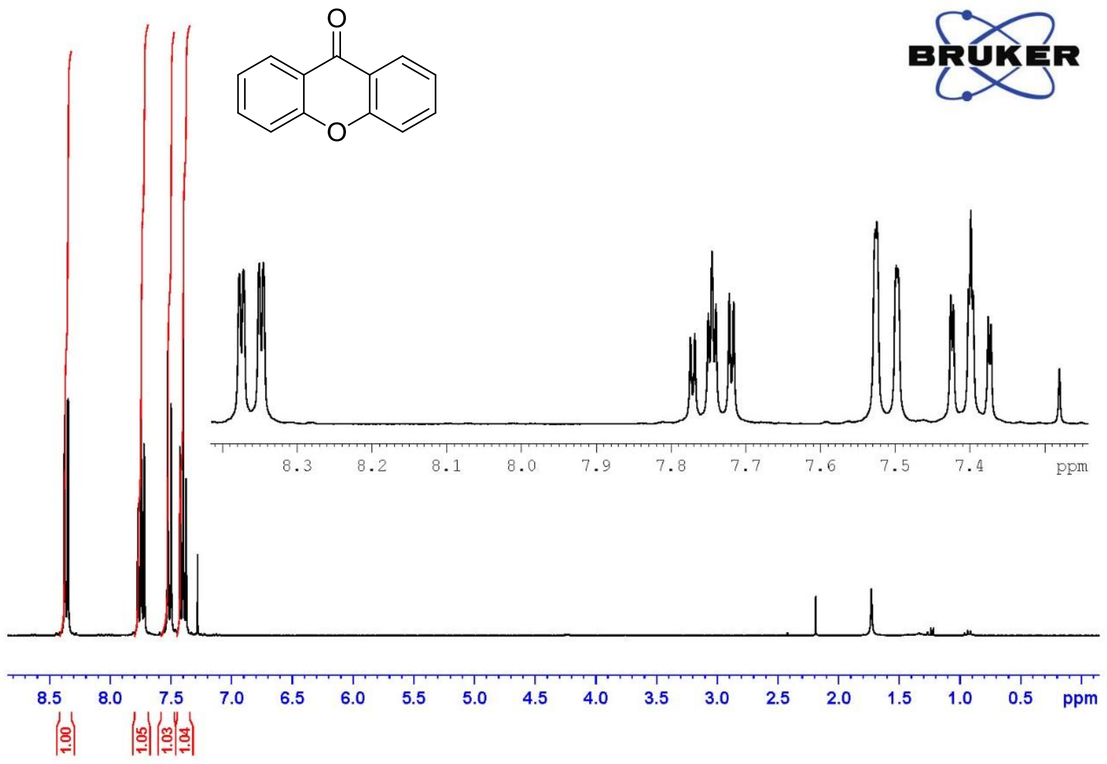

Figure S29. ^{1}H NMR of xanthone (**5a**).

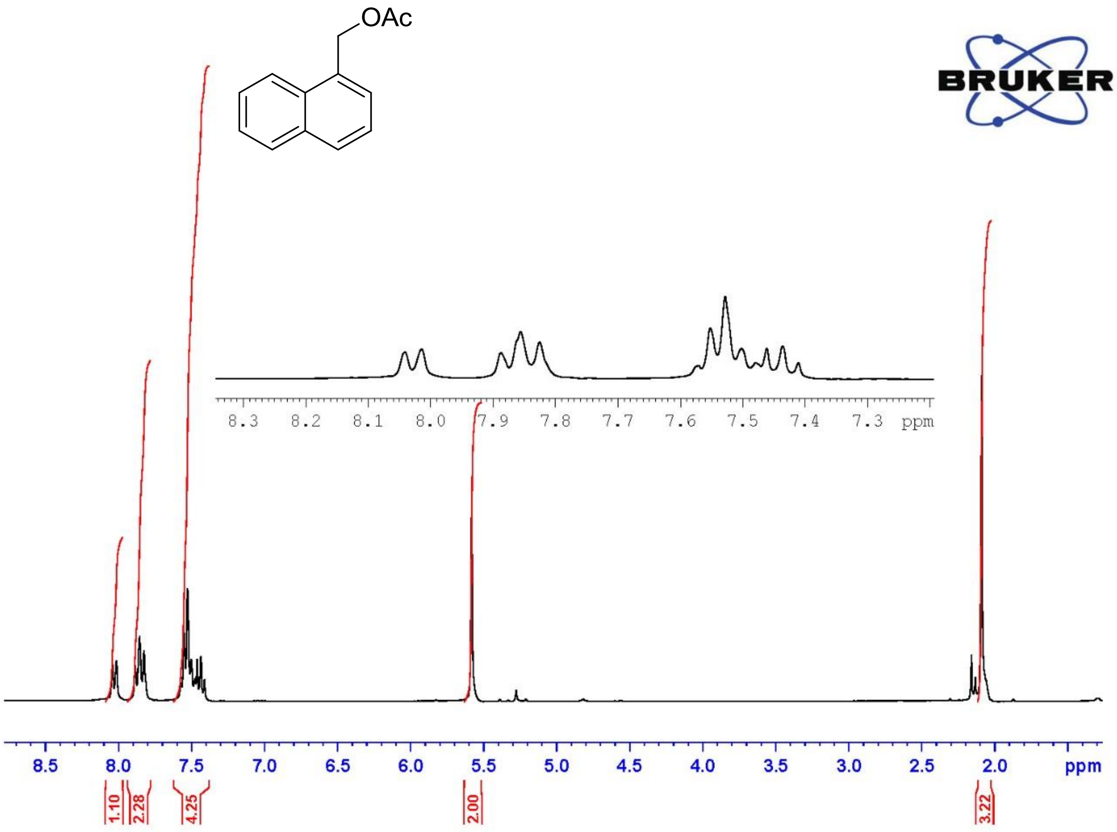

Figure S30. ^{1}H NMR of naphthalene-1-ylmethyl acetate (**7a**).

References

1. Alaimo, R. J., Handbook of chemical health and safety, American Chemical Society ; Oxford University Press: Washington, D.C.; Oxford [England]; New York, 2001.
2. Adams, H.; Bawa, R. A.; Jones, S., *Organic & Biomolecular Chemistry* **2006,** *4* (22), 4206-4213.
3. Sugamoto, K.; Matsushita, Y.-i.; Kameda, Y.-h.; Suzuki, M.; Matsui, T., *Synth. Commun.* **2005,** *35* (1), 67-70.
4. Zhang, Q.; Chen, C.; Ma, H.; Miao, H.; Zhang, W.; Sun, Z.; Xu, J., *Journal of Chemical Technology & Biotechnology* **2008,** *83* (10), 1364-1369.
5. Zhou, M.; Li, X.; Bao, L.; Yuan, X.; Luo, H. a., *Catal. Lett.* **2016,** *146* (2), 383-390.
6. Chanmiya Sheikh, M.; Takagi, S.; Ogasawara, A.; Ohira, M.; Miyatake, R.; Abe, H.; Yoshimura, T.; Morita, H., *Tetrahedron* **2010,** *66* (12), 2132-2140.
7. Rueda-Becerril, M.; Chatalova Sazepin, C.; Leung, J. C. T.; Okbinoglu, T.; Kennepohl, P.; Paquin, J.-F.; Sammis, G. M., *J. Am. Chem. Soc.* **2012,** *134* (9), 4026-4029.
8. Baba, H.; Moriyama, K.; Togo, H., *Tetrahedron Lett.* **2011,** *52* (33), 4303-4307.
9. Magens, S.; Ertelt, M.; Jatsch, A.; Plietker, B., *Org. Lett.* **2008,** *10* (1), 53-56.
10. Powell, D. A.; Pelletier, G., *Tetrahedron Lett.* **2008,** *49* (16), 2495-2498.
11. Savile, C. K.; Kazlauskas, R. J., *J. Am. Chem. Soc.* **2005,** *127* (35), 12228-12229.
12. Rodriguez, A.; Moran, W. J., *Eur. J. Org. Chem.* **2009,** *2009* (9), 1313-1316.
13. Mino, T.; Hasegawa, T.; Shirae, Y.; Sakamoto, M.; Fujita, T., *J. Organomet. Chem.* **2007,** *692* (20), 4389-4396.
14. Kesharwani, T.; Larock, R. C., *Tetrahedron* **2008,** *64* (26), 6090-6102.
15. Kim, M.-J.; Choi, Y. K.; Kim, S.; Kim, D.; Han, K.; Ko, S.-B.; Park, J., *Org. Lett.* **2008,** *10* (6), 1295-1298.
16. Zhao, J.; Larock, R. C., *Org. Lett.* **2005,** *7* (19), 4273-4275.
17. Wang, Y.; Zhang, L.; Yang, Y.; Zhang, P.; Du, Z.; Wang, C., *J. Am. Chem. Soc.* **2013,** *135* (48), 18048-18051.
